# CD38 deficiency alleviates Ang II-induced vascular remodeling by inhibiting small extracellular vesicle-mediated vascular smooth muscle cell senescence in mice

**DOI:** 10.1038/s41392-021-00625-0

**Published:** 2021-06-11

**Authors:** Lu Gan, Demin Liu, Jing Liu, Erya Chen, Chan Chen, Lian Liu, Hang Hu, Xiaohui Guan, Wen Ma, Yanzi Zhang, Yarong He, Bofu Liu, Songling Tang, Wei Jiang, Jianxin Xue, Hongbo Xin

**Affiliations:** 1 Research Laboratory of Emergency Medicine, Department of Emergency Medicine, National Clinical Research Center for Geriatrics, West China Hospital, Sichuan University, Chengdu, People’s Republic of China; 2 Cardiology Department, The Second Hospital of Hebei Medical University, Shijiazhuang, People’s Republic of China; 3 Department of Endocrinology, The Second Hospital of Shanxi Medical University, Taiyuan, People’s Republic of China; 4 Department of Anesthesiology, Laboratory of Anesthesia and Critical Care Medicine, Translational Neuroscience Center, West China Hospital, Sichuan University, Chengdu, People’s Republic of China; 5 The National Engineering Research Center for Bioengineering Drugs and the Technologies, Institute of Translational Medicine, Nanchang University, Nanchang, People’s Republic of China; 6 State Key Laboratory of Biotherapy, West China Hospital, Sichuan University, Chengdu, People’s Republic of China; 7 Department of Thoracic Oncology, Cancer Center, West China Hospital, Sichuan University, Chengdu, People’s Republic of China

**Keywords:** Cardiovascular diseases, Senescence

## Abstract

CD38 is the main enzyme for nicotinamide adenine dinucleotide (NAD) degradation in mammalian cells. Decreased NAD levels are closely related to metabolic syndromes and aging-related diseases. Our study showed that CD38 deficiency significantly alleviated angiotensin II (Ang II)-induced vascular remodeling in mice, as shown by decreased blood pressures; reduced vascular media thickness, media-to-lumen ratio, and collagen deposition; and restored elastin expression. However, our bone marrow transplantation assay showed that CD38 deficiency in lymphocytes led to lack of protection against Ang II-induced vascular remodeling, suggesting that the effects of CD38 on Ang II-induced vascular remodeling might rely primarily on vascular smooth muscle cells (VSMCs), not lymphocytes. In addition, we observed that CD38 deficiency or NAD supplementation remarkably mitigated Ang II-induced vascular senescence by suppressing the biogenesis, secretion, and internalization of senescence-associated small extracellular vesicles (SA-sEVs), which facilitated the senescence of neighboring non-damaged VSMCs. Furthermore, we found that the protective effects of CD38 deficiency on VSMC senescence were related to restoration of lysosome dysfunction, particularly with respect to the maintenance of sirtuin-mediated mitochondrial homeostasis and activation of the mitochondria–lysosomal axis in VSMCs. In conclusion, our findings demonstrated that CD38 and its associated intracellular NAD decline are critical for Ang II-induced VSMC senescence and vascular remodeling.

## Introduction

The incidence of multiple chronic “burdens of lifestyle” diseases, such as cardiovascular diseases (CVDs), is significantly increased with aging.^1^ The accumulation of senescent cells, as the main event in aging,^2^ has been demonstrated to be involved in the pathological progression of CVDs, including heart failure,^3^ atherosclerotic disorder,^4^ and hypertension.^5^ The senescence of vascular smooth muscle cells (VSMCs) contributes to pathological vascular remodeling and the deterioration of hypertension control.^6,7^

Nicotinamide adenine dinucleotide (NAD), as a cofactor or substrate of numerous key intracellular enzymes, participates in multiple physiological and pathological processes, particularly in aging. Considerable evidence has demonstrated that a decline in intracellular NAD levels accelerates cell senescence and induces organ function disorders.^8,9^ CD38 is a transmembrane protein with both ADP-ribosyl cyclase and cyclic ADP ribose hydrolase activities for which NAD is a substrate for generating second messengers in intracellular calcium signaling.^10^ Emerging evidence indicates that CD38, as the main NAD-degradation enzyme (NADase) in mammalian cells, is involved in various physiological and pathological processes by regulating intracellular NAD levels.^10^ Considering the significant roles of NAD metabolism in the aging process, the effects and underlying mechanisms of CD38-mediated intracellular NAD decline in age-related diseases have drawn a great deal of attention.^11–13^

Small extracellular vesicles (sEVs), including exosomes, deliver signaling molecules for intercellular and interorgan communication.^14,15^ Recently, sEVs have been described as senescence-associated secretory mediators^16–18^ that alter the microenvironment and promote neighboring cell senescence in aging processes.^19–21^ Moreover, CD38 expression is upregulated by the senescence-associated secretory phenotype (SASP) effect, linking senescence and cellular NAD decline.^22^ Latifkar et al.^23^ reported that Sirtuin 1 (SIRT1), a deacetylase for which NAD is a substrate, inhibited breast cancer invasion through the regulation of exosome secretion by controlling lysosomal acidification, suggesting that CD38 may be involved in the biogenesis, secretion, and paracrine effects of senescence-associated small extracellular vesicles (SA-sEVs).

Therefore, we speculate that CD38 deficiency may alleviate vascular remodeling in hypertension by inhibiting VSMC senescence. In the present study, we observe that CD38 deficiency significantly attenuated Ang II infusion-induced hypertension and vascular remodeling by delaying VSMC senescence. Furthermore, we provide strong evidence that CD38 deficiency or NAD supplementation markedly alleviated VSMC senescence by suppressing the biogenesis, secretion, and internalization of SA-sEVs in VSMCs, suggesting that “NAD-boosting” therapy and/or the application of CD38 inhibitors may be novel strategies for treating age-related diseases by suppressing VSMC senescence.

## Results

### CD38 deficiency alleviated Ang II-induced hypertension and vascular remodeling

**Fig. 1:**
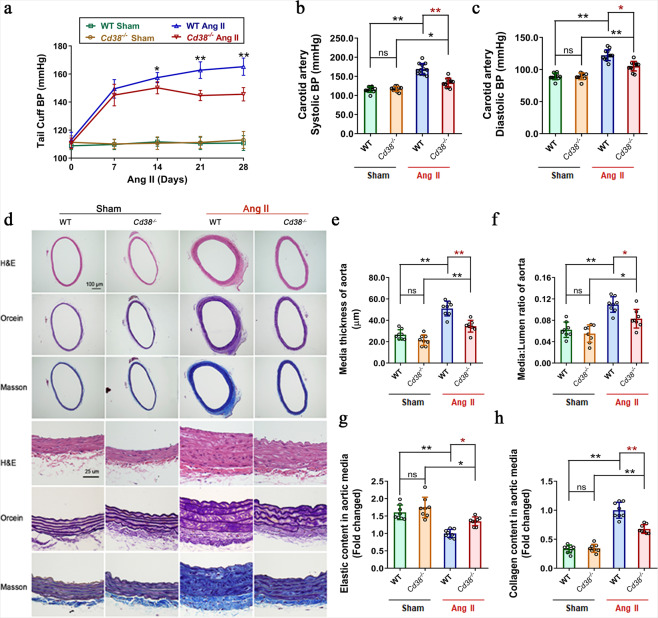
CD38 deficiency alleviated Ang II infusion-induced hypertension and vascular remodeling. **a** Caudal artery blood pressures were detected in *Cd38*^*−/−*^ and WT mice every 7 days after Ang II or saline infusion (*n* ≥ 10, two-way ANOVA, *p < 0.05, **p < 0.01). **b**, **c** The systolic (**b**) and diastolic (**c**) blood pressures of the carotid artery were detected in *Cd38*^*−/−*^ and WT mice 4 weeks after Ang II or saline infusion. (*n* ≥ 10, one-way ANOVA, *p < 0.05, **p < 0.01). **d** Vascular remodeling was analyzed in thoracic aorta sections obtained from WT and *Cd38*^*−/−*^ mice with or without Ang II stimulation. Representative images of vessel sections stained with H&E, Orcein staining (the dark reddish-brown area represented elastin), and Masson trichrome blue staining (blue represents collagen deposition). **e**–**h** Medium thickness (**e**) and median-to-lum**e**n ratio (**f**) of the aortas were calculated on the basis of the H&E staining, and the density of stained elastin (**g**) and collagen (**h**) in the aortic smooth muscle wall were quantitatively analyzed by Orcein staining and Masson trichrome blue staining, respectively (*n* = 8, one-way ANOVA, *p < 0.05, **p < 0.01)

To investigate the role of CD38 in hypertension and hypertension-induced vascular remodeling, mouse models of hypertension were established by administrating Ang II infusion (490 ng/min/kg) subcutaneously through osmotic pumps for 4 weeks. As shown in Fig. 1a, the Ang II-induced elevation in caudal arterial pressure was significantly alleviated in *Cd38*-knockout (*Cd38*^*−/−*^) mice compared with wild-type (WT) mice, whereas there was no difference in caudal arterial pressure between *Cd38*^*−/−*^ and WT mice with vehicle infusion (sham group). Furthermore, the Ang II-induced increases in systolic blood pressure (SBP) and diastolic blood pressure (DBP) measured in the carotid artery were dramatically decreased, by 20.99% (Figs. 1b) and 13.92% (Fig. 1c), in the *Cd38*^*−/−*^ mice compared with that in the WT mice, respectively. Interestingly, CD38 expression was significantly upregulated by Ang II stimulation in the aortic artery, as detected by immunochemistry and immunofluorescent staining (Supplementary Fig. S1). Considering the exact regulatory effects of CD38 on respiratory smooth muscle cells in asthma shown in previous studies,^24,25^ the elevation of CD38 expression in the media layer of the aorta in the present study suggested that CD38 may have the potential to regulate the pathological changes of VSMCs in hypertension.

To assess the effects of *Cd38* gene deletion on vascular remodeling induced by Ang II stimulation, in this study, the media thickness, media-to-lumen ratio of aortas and the expression of elastic fiber and collagen were determined by H&E, Orcein, and Masson staining (Fig. 1d). After 4 weeks of Ang II infusion, the vascular media thickness and the media-to-lumen ratio in the *Cd38*^*−/−*^ mice were significantly reduced, by 28.65% (Fig. 1e) and 21.36% (Fig. 1f), respectively, compared to the WT mice. Moreover, CD38 deficiency also attenuated Ang II-induced collagen deposition (30.04%, Fig. 1h) and restored elastin expression (31.45%, Fig. 1g). Similar results were obtained in the smaller artery, the mesenteric artery, indicated by the reduced vascular media thickness (26.64%, Supplementary Fig. S2b) and the media-to-lumen ratio (43.79%, Supplementary Fig. S2c), attenuated collagen deposition (37.18%, Supplementary Fig. S2e) and restored elastin expression (26.24%, Supplementary Fig. S2d) in Ang II *Cd38*^*−/−*^ mice compared with Ang II WT mice (Supplementary Fig. S2). Taken together, these results demonstrated that CD38 deficiency ameliorated Ang II-induced hypertension and hypertension-induced vascular remodeling.

Considering the importance of CD38 in lymphocytes, the effects of normal CD38-expressing lymphocytes on Ang II-induced hypertension and vascular remodeling were evaluated with a bone marrow transplantation assay (Supplementary Fig. S3a). Unexpectedly, compared to those in WT mice with WT bone marrow transplants (WT (WT BM)), the Ang II-induced alterations in blood pressures (Supplementary Fig. S3b–d) and aortal structures (Supplementary Fig. S3e–i) were restored in *Cd38*^*−/−*^ mice with WT bone marrow transplants (*Cd38* (WT BM)), indicating that CD38 deficiency-mediated protective effects on Ang II-induced hypertension and vascular remodeling might rely primarily on the regulation of the functions of vascular structural cells. In addition, our results also showed no significant differences in aortic media, media-to-lumen ratio, extensive collagen deposition or elastin expression after Ang II administration in WT mice and WT or *Cd38*^*−/−*^ bone marrow-transplanted mice, further confirming that CD38 deficiency in bone marrow-derived lymphocytes did not contribute to the alleviation of Ang II-induced hypertension and vascular remodeling in mice.

### CD38 deficiency ameliorated Ang II-induced smooth muscle cell senescence in a NAD-dependent manner

**Fig. 2:**
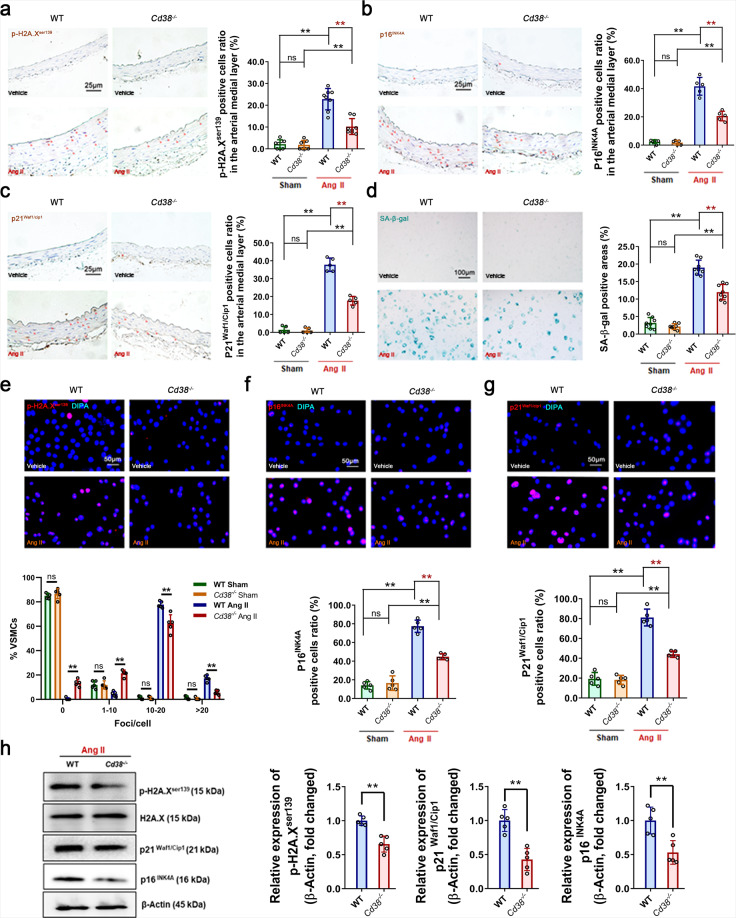
CD38 deficiency alleviated Ang II-induced VSMC senescence. **a**–**c** The accumulation of the senescence marker protein p-H2A.X^ser139^ (**a**), p21^Waf1/Cip1^ (**b**), and p16^INK4A^ (**c**) was analyzed by immunohistochemistry staining of the mouse arterial medial layer with Ang II or saline infusion. The dark reddish-brown dots represented the accumulation of senescence marker proteins in the VSMC nucleus (*n* = 8, unpaired *t* test, *p < 0.05, **p < 0.01). **d** SA-β-gal staining showed VSMC senescence with Ang II or Vehicle administration. (*n* = 8, one-way ANOVA, *p < 0.05, **p < 0.01). **e**–**g** The expression of the senescence marker protein p-H2A.X^ser139^ (**e**), p21^Waf1/Cip1^ (**f**), and p16^INK4A^ (**g**) was analyzed by immunofluorescence staining in Ang II-challenged VSMCs. (*n* = 8, unpaired *t*-test, *p < 0.05, **p < 0.01). **h** The expression of senescence-associated proteins in VSMCs with Ang II administration was detected by western blot assay (*n* = 5, unpaired *t*-test, *p < 0.05, **p < 0.01). Ang II (100 μM) was added to the cell medium for 3 days, the senescent cells were stained, and the protein levels were analyzed

It has been reported that vascular smooth muscle cell (VSMC) senescence is closely related to Ang II-induced hypertension and vascular remodeling.^6,7^ To clarify whether VSMC senescence was involved in the protective effect of CD38 deficiency on Ang II-induced vascular remodeling in the present study, the effects of Ang II stimulation on VSMC senescence were determined. It has been reported that β-galactosidase (SA-β-gal), a lysosomal enzyme, was utilized to detect senescent cells,^26^ and the CDK2 inhibitor p21^Waf1/Cip1^ (CDKN1A) and CDK4/6 inhibitor p16^INK4A^ (CDKN2A) accumulated with aging.^27^ The phosphorylation of nuclear histone H2A.X (p-H2A.X) is representative of DNA damage response (DDR)-dependent senescence^26^ and was previously shown to be significantly elevated in Ang II-stimulated smooth muscle cells.^28^ Our immunohistochemistry staining results demonstrated that CD38 deficiency significantly attenuated Ang II-induced DNA damage (p-H2A.X^ser139^, 44.81%, Fig. 2a) and reduced the accumulation of senescence-associated markers p21^Waf1/Cip1^ (50.57%, Fig. 2b) and p16^INK4A^ (53.15%, Fig. 2c) in VSMCs in the mouse arterial medial layer. In addition, SA-β-gal staining of primary mouse VSMCs revealed that CD38 deficiency remarkably alleviated Ang II-induced VSMC senescence, by 36.94% (Fig. 2d). These results were confirmed by an immunofluorescent staining assay (Fig. 2e–g) and western blot analysis (Fig. 2h) with primary mouse VSMCs, which revealed decreases in p-H2A.X^ser139^-, p21^Waf1/Cip1^- and p16^INK4A^-positive cells and their protein expression in CD38-deficient mice challenged with Ang II.

**Fig. 3:**
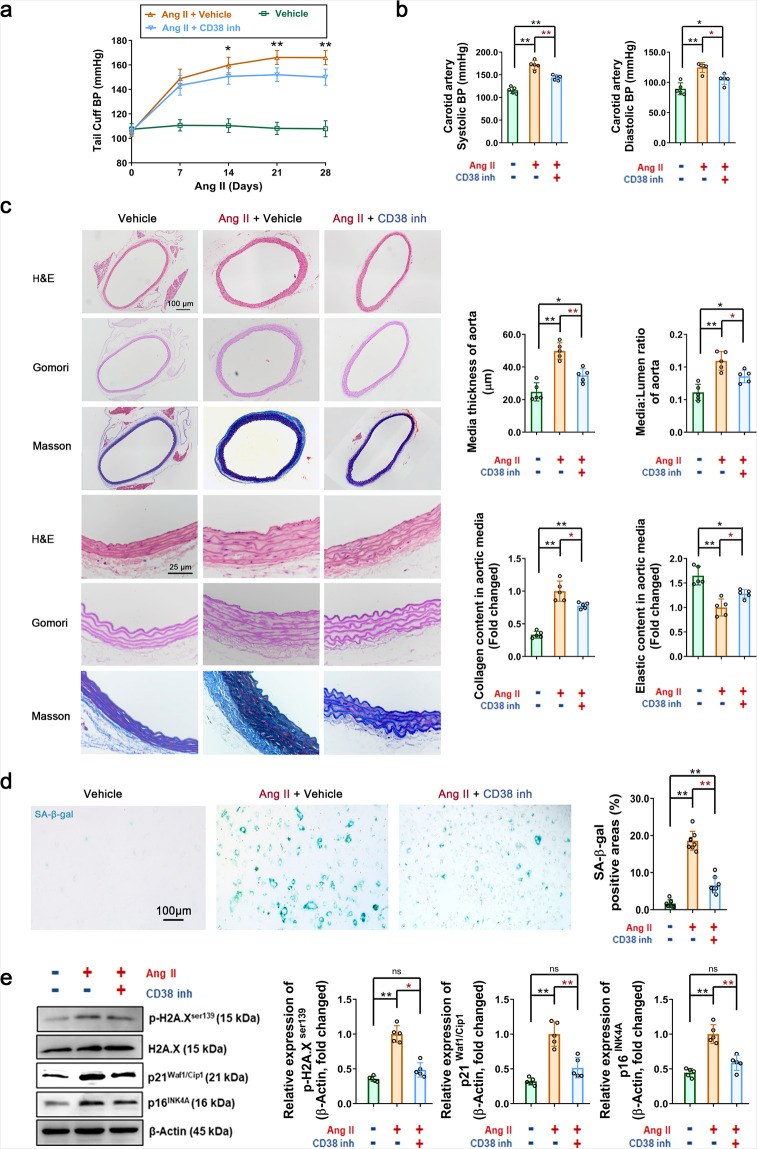
Inhibition of CD38 activities alleviated Ang II-induced hypertension, vascular remodeling, and VSMC senescence. **a** Caudal artery blood pressures were detected in WT mice with CD38 inhibitor (inh) or Vehicle administration every 7 days after Ang II infusion (*n* = 10, two-way ANOVA, *p < 0.05, **p < 0.01). **b** The systolic and diastolic blood pressures of the carotid artery were detected in WT mice with CD38 inhibitor or Vehicle administration 4 weeks after Ang II infusion (*n* = 10, one-way ANOVA, *p < 0.05, **p < 0.01). **c** Vascular remodeling was analyzed in thora**c**ic aorta sections from WT mice with or without Ang II + CD38 inhibitor treatment. Representative images of vessel sections stained with H&E, Gomori’s aldehyde-fuchsin stain (bright purple represented elastin), and Masson trichrome blue stain (blue represented collagen deposition). The median thickness and median-to-lumen ratio of the aortas were calculated by H&E staining, and the density of stained elastin and collagen in the aortic smooth muscle wall was quantitatively analyzed by Gomori’s aldehyde-fuchsin staining and Masson trichrome blue staining, respectively (*n* = 5, one-way ANOVA, *p < 0.05, **p < 0.01). **d**, **e** CD38 inhibitor restored Ang II-induced cell senescence. VSMCs were pretreated with CD38 inhibitor for 4 h prior to Ang II administration. All these compounds were maintained in the medium for 3 days. Then, the VSMCs were stained with (**d**) SA-β-gal, and (**e**) the proteins were analyzed by western blot analysis (*n* ≥ 5, one-way ANOVA, *p < 0.05, **p < 0.01)

To validate the regulatory role of CD38 in senescence, compound 78c^29^ was applied as a CD38-specific inhibitor (CD38 inh). The results showed that following chronic administration of 78c, the caudal arterial pressure, systolic blood pressure, and diastolic blood pressure were significantly reduced (Fig. 3a). The vascular media thickness and the media-to-lumen ratio in 78c-administered mice were significantly reduced, by 30.02% and 21.57% (Fig. 3b), respectively. Moreover, 78c also reduced Ang II-induced collagen deposition (Fig. 3b, 22.20%) and restored elastin expression (Fig. 3b, 28.06%). In vitro, 78c (CD38 inh, 0.5 μM) also significantly alleviated Ang II-induced VSMC senescence, as shown by the 64.58% reduction in the SA-β-gal-positive area (Fig. 3c) and decreased protein levels of p-H2A.X^ser139^, p21^Waf1/Cip1^, and p16^INK4A^ (by 53.50%, 48.40%, and 41.37%, respectively, Fig. 3d). CD38, as a main NADase in mammalian cells, is critical for age-related NAD decline.^11^ CD38 requires not only intracellular NAD but also circulating NAD precursors such as nicotinamide mononucleotide (NMN) and nicotinamide riboside (NR), which are incorporated inside cells and consumed by NAD biosynthetic pathways. Therefore, we also examined whether NAD supplementation can rescue Ang II-induced smooth muscle cell senescence. Studies have shown that intracellular NAD levels are significantly increased in multiple tissues of *Cd38*^*−/−*^ mice.^15^ As shown in Supplementary Fig. S5a, under physiological conditions, the intracellular NAD levels of whole aorta tissue lysates were increased by 48.47% in *Cd38*^*−/−*^ mice compared with WT mice. Moreover, in vivo oral administration of NMN to elevate NAD levels alleviated Ang II-induced blood pressure elevation (Supplementary Fig. S4a, b). In Ang II-challenged mice, the vascular media thickness, media-to-lumen ratio, and collagen deposition were reduced by 26.19%, 27.05%, and 30.40%, respectively, following intragastric administration of NMN, and elastin expression was restored by 24.38% after NMN treatment (Supplementary Fig. S4c). In vitro, we pre-administered exogenous NAD (100 μM) to WT VSMCs and detected indicators of Ang II-induced cell senescence. The results showed that the SA-β-gal-positive area was decreased by 54.54% (Fig. 3c), and the protein levels of p-H2A.X^ser139^, p21^Waf1/Cip1^, and p16^INK4A^ were downregulated by 42.89%, 38.92%, and 53.40%, respectively (Fig. 3d). In addition, SA-β-gal staining analysis showed that overexpression of CD38 significantly promoted the senescence of WT VSMCs (Supplementary Fig. S5b). These results strongly supported that CD38 may be a potential pharmacological target for anti-senescence treatment.

### CD38 deficiency delayed cell senescence by inhibiting the generation and secretion of senescence-associated small extracellular vesicles (SA-sEVs)

**Fig. 4:**
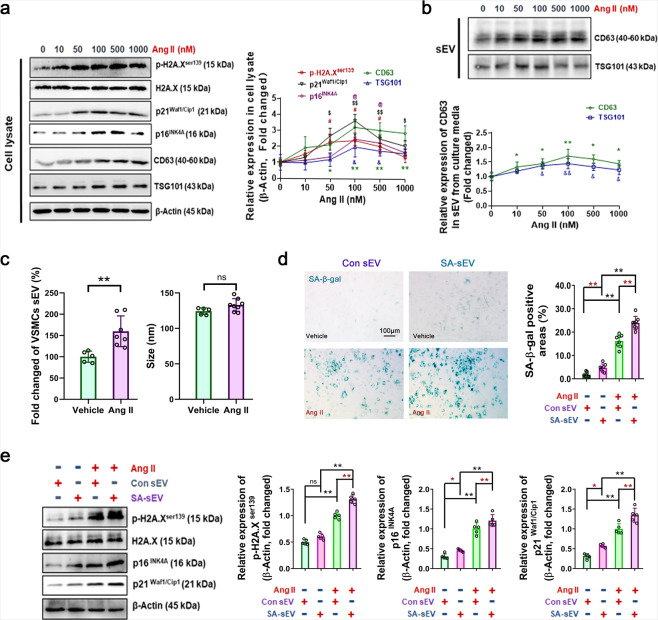
Excess SA-sEVs was generated in senescent VSMCs and promote cell senescence under Ang II administration. **a**, **b** In vitro sEV biogenesis analysis in VSMCs. Three days after incubation with different concentrations of Ang II, the expression of senescence marker proteins (p-H2A.X^ser139^, p21^Waf1/Cip1^, and p16^INK4A^) and sEV (exosome) marker proteins (CD63 and TSG101) in cell lysates and sEVs, respectively, were analyzed by western blot assay (*n* ≥ 5, two-way ANOVA, *p < 0.05, **p < 0.01). **c** The quantity and size of the sEVs derived from VSMCs were analyzed by NanoSight tracking assay. sEVs were extracted from VSMC culture medium and analyzed by a NS300 instrument (*n* ≥ 5, one-way ANOVA, *p < 0.05, **p < 0.01). **d**, **e** SA-sEVs promoted Ang II-induced senescence. VSMCs were treated overnight with SA-sEVs derived from senescent cells. Then, Ang II (100 nM) was added and maintained for 3 days. VSMC senescence was analyzed by (**d**) SA-β-gal staining and (**e**) western blot analysis of aging-related proteins. The cells were stained and the proteins were analyzed (*n* ≥ 5, one-way ANOVA, *p < 0.05, **p < 0.01)

Evidence has indicated that small extracellular vesicles (sEVs), including exosomes, facilitate neighboring nondamaged cell senescence via a bystander effect.^30^ We, therefore, explored the effects of CD38 deficiency on Ang II-induced generation of sEVs. Our results showed that Ang II promoted the expression of the exosome protein markers CD63 and TSG101 in cell lysates and sEV pellets, and elevated p-H2A.X^ser139^, p21^Waf1/Cip1^, and p16^INK4A^ expression (Fig. 4a, b). First, VSMC-derived sEVs were identified by western blot, NanoSight tracing analysis, and transmission electron microscopy (TEM), in which sEVs were positive for exosome protein markers (CD63 and CD81) with approximate diameters <300 nm and typical morphological characteristics (Supplementary Fig. S6). In addition, NanoSight tracing analysis showed that VSMCs challenged with Ang II were able to release more small particles (increased by 59.78%, Fig. 4c), indicating that Ang II was able to promote sEV generation by senescent cells. Furthermore, we sought to determine whether sEVs derived from Ang II-challenged VSMCs, which were called senescence-associated sEVs (SA-sEVs), can promote cell senescence (Fig. 4d, e). Our results demonstrated that SA-sEVs accelerated VSMC senescence in both the Ang II administration and control groups. Furthermore, compared to sEVs secreted by control VSMCs (Con-sEV), SA-sEVs increased the SA-β-gal-positive area by 47.91% (Fig. 4d) and increased the expression of the aging-related protein p-H2A.X^ser139^, p21^Waf1/Cip1^, and p16^INK4A^ by 31.20%, 42.44%, and 35.92% in Ang II-challenged VSMCs, respectively (Fig. 4e).

**Fig. 5:**
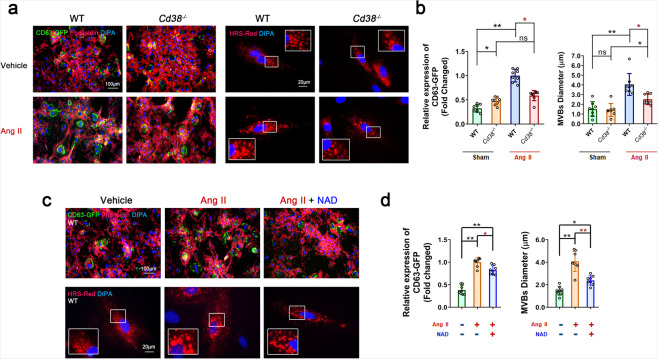
CD38 deficiency and NAD supplementation suppressed sEV biogenesis by inhibiting MVB formation. **a**, **b** The numbers of CD63-GFP-positive exosomes and HRS-RFP-positive MVBs were reduced in *Cd38*^*−/−*^ VSMCs stimulated with Ang II for 3 days. Phalloidin was used to show the cytoskeleton in red. (*n* ≥ 5, one-way ANOVA, *p < 0.05, **p < 0.01). **c**, **d** The numbers of CD63-GFP+ exosomes and HRS-RFP+ MVBs were reduced by NAD supplementation coupled with Ang II stimulation for 3 days (*n* ≥ 5, one-way ANOVA, *p < 0.05, **p < 0.01)

Multivesicular bodies (MVBs), the specific endosomes involved in exosome secretion, are regulated by hepatocyte growth factor-regulated tyrosine kinase substrate (HRS), which is often used as an MVB marker. In the present study, VSMCs stably overexpressing exosomal Cyto-Tracer CD63-GFP fusion protein and VSMCs presenting HRS-RFP-positive (HRS-RFP+) MVBs after transfection with the pCS2 HRS-RFP plasmid were prepared. As shown in Fig. 5a, c, the number of CD63-GFP-positive (CD63-GFP+) dots were markedly increased and accumulated in Ang II-challenged VSMCs, indicating that CD63-GFP+sEVs were highly produced in senescent VSMCs. As shown in Fig. 5a, c, the HRS-RFP+MVBs were enlarged and increased in number after Ang II stimulation of VSMCs, compared to those in the vehicle group. In addition, the Ang II-induced alterations of CD63-GFP+ and HRS-RFP+ structures were attenuated in *Cd38*^*−/−*^ VSMCs (Fig. 5a, b) and in WT VSMCs treated with NAD (Fig. 5c, d). Moreover, CD63 expression was elevated in the p16^INK4A^-positive VSMCs of Ang II-infused mice, and its expression was attenuated in the VSMCs of *Cd38*^*−/−*^ mice (Supplementary Fig. S7a) or WT mice after NMN administration (Supplementary Fig. S7b). All the results suggested that either CD38 deficiency or NAD supplementation might have inhibited cell senescence by suppressing biogenesis and secretion, which were represented as CD63 accumulation and MVB formation, respectively.

### CD38 deficiency inhibited Ang II-induced sEV biogenesis by re-establishing the mitochondrial-lysosomal axis in a NAD/sirtuin-dependent manner

**Fig. 6:**
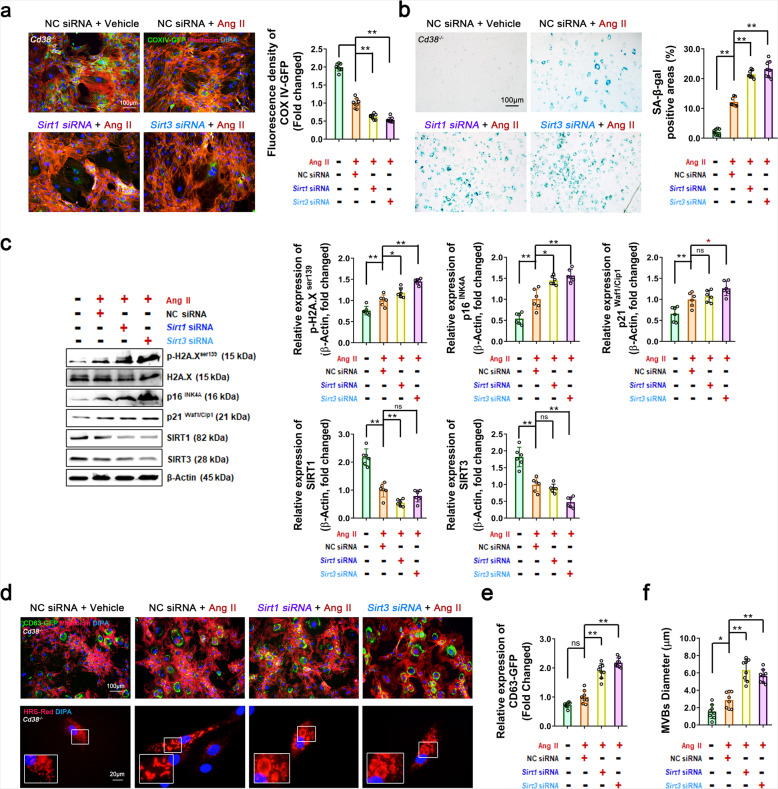
CD38 deficiency-mediated SIRT1/SIRT3 activation alleviated VSMC senescence by inhibiting the biogenesis and secretion of SA-sEVs. **a** In vitro mitochondria quantity analysis. The mitochondrial mass was analyzed in stable *Cd38*^−*/−*^ COX IV-GFP VSMCs. VSMCs were transfected with *Sirt1* and *Sirt3* siRNAs and negative control (NC) siRNA. After transfection overnight, Ang II (100 nM) was added and maintained for 3 days. The mitochondria were tracked using green fluorescent dots (*n* ≥ 5, one-way ANOVA, *p < 0.05, **p < 0.01). **b**, **c** In vitro VSMC senescence analysis. VSMC senescence was analyzed by SA-β-gal staining (**b**) and western blot analysis of aging-related proteins (**c**) (*n* ≥ 5, one-way ANOVA, *p < 0.05, **p < 0.01). The *Cd38*^−*/−*^ VSMCs were transfected with siRNA. After transfection overnight, Ang II (100 nM) was added and maintained for 3 days. Then, the total proteins were extracted for analysis. **d**–**f** In vitro sEV biogenesis analysis. CD63-GFP+ exosomes and HRS-RFP+ MVBs were analyzed in stable *Cd38*^−*/−*^ CD63-GFP VSMCs and *Cd38*^−*/−*^ VSMCs. VSMCs were transfected with siRNA or siRNA plus pCS2 HRS-RFP plasmids. After transfection overnight, Ang II (100 nM) was added and maintained for 3 days. The exosomes and MBVs were tracked by green fluorescence and red fluorescence, respectively (*n* ≥ 5, one-way ANOVA, *p < 0.05, **p < 0.01)

Sirtuins, as NAD-dependent deacetylases, have been demonstrated to play key roles in extending life span and preventing age-related diseases. Camacho-Pereira and colleagues^11^ demonstrated that CD38-induced age-related NAD decline was correlated with the development of mitochondrial dysfunction. Mitochondrial dysfunction is a hallmark of aging.^31^ Moreover, SIRT1^32^ and SIRT3^11^ have shown prominent protective effects against senescence-associated mitochondrial dysfunction and prevent aging by maintaining mitochondrial homeostasis. In the present study, *Sirt1* and *Sirt3* overexpression (*Sirt1* OE and *Sirt3* OE) significantly reduced p-H2A.X^ser139^, p21^Waf1/Cip1^, and p16^INK4A^ protein levels in WT VSMCs after Ang II administration, suggesting that SIRT1 and SIRT3 activation alleviated Ang II-induced VSMC senescence (Supplementary Fig. S8a). In contrast, inhibition of SIRT1 or SIRT3 exacerbated Ang II-induced cell senescence. As shown in Fig. 6a, the expression of mitochondrial Cyto-Tracer (pCT-Mito-GFP (pCMV with COX IV Tag)) was markedly decreased upon suppression of SIRT1 and SIRT3 expression with specific siRNAs in *Cd38*^*−/−*^ VSMCs, suggesting that the mitochondrial mass was significantly decreased. In addition, the SA-β-gal-positive areas (Fig. 6b) and the expression of senescence marker proteins (Fig. 6c) were remarkably increased by inhibition of SIRT1 and SIRT3 in *Cd38*^*−/−*^ VSMCs, indicating that the CD38 deficiency-triggered inhibition of Ang II-induced senescence was dependent on SIRT1 and SIRT3 activation. Furthermore, Ang II-induced accumulation of CD63-GFP+sEVs and the enlargement and increase in HRS-RFP+MVBs were reversed by *Sirt1* OE and *Sirt3* OE in WT VSMCs (Supplementary Fig. S8b). In contrast, Ang II-mediated alterations were aggravated by SIRT1 or SIRT3 inhibition in *Cd38*^*−/−*^ VSMCs (Fig. 6d–f), suggesting that Ang II contributed to SA-sEV biogenesis, which relied on CD38 and resulted in the inactivation of SIRT1 and SIRT3.

**Fig. 7:**
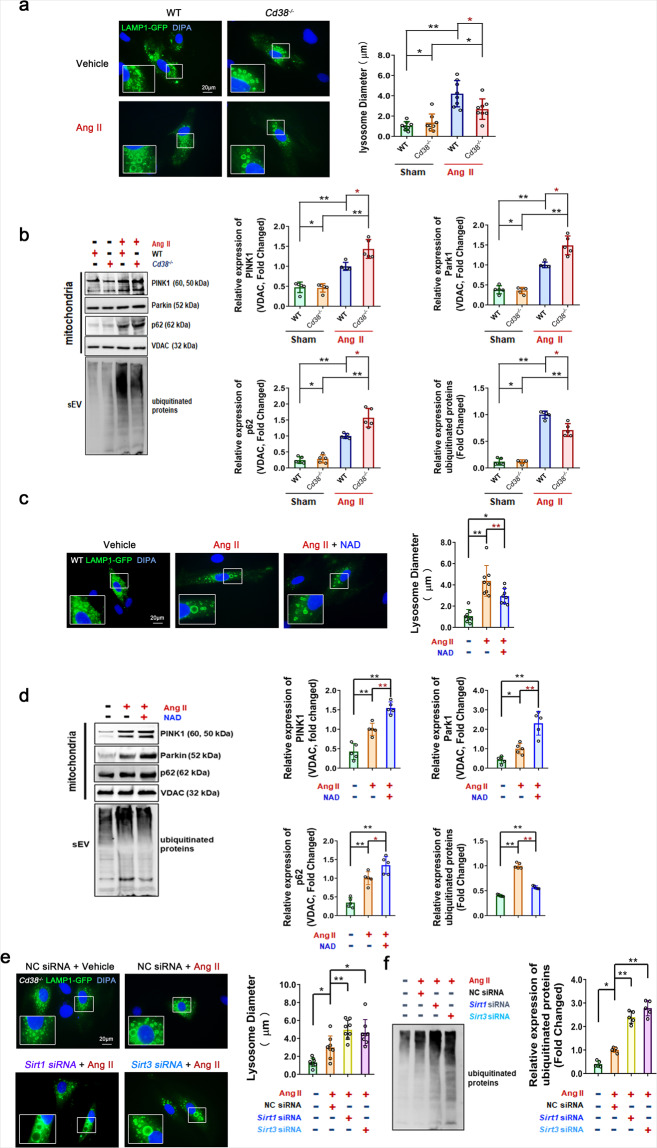
CD38 deficiency prevented SA-sEV biogenesis in VSMCs by reestablishing the mitochondrial-lysosomal axis in a NAD/Sirtuin-dependent manner. **a**, **b** In vitro lysosome function and mitophagy analysis in VSMCs with or without CD38 deficiency. **a** WT and *Cd38*^−*/−*^ VSMCs were transfected overnight with the LAMP1-mGFP plasmid and treated with or without Ang II (100 nM) for 3 days. The green fluorescing structures were tracked to lysosomes (*n* ≥ 5, one-way ANOVA, *p < 0.05, **p < 0.01). **b** The expression of mitophagy-associated proteins (PINK1, Parkin and p62) in mitochondria and ubiquitinated proteins in sEVs were analyzed by western blot assay. WT and *Cd38*^−*/−*^ VSMCs were treated with or without Ang II (100 nM) for 3 days (*n* ≥ 5, one-way ANOVA, *p < 0.05, **p < 0.01). **c**, **d** In vitro lysosome function and mitophagy analysis in WT VSMCs treated with NAD supplementation. VSMCs were transfected with LAMP1-mGFP plasmids and then treated with or without NAD (100 μM) plus Ang II (100 nM). **c** Lysosomal structures were detected by cellular microfluorescence imaging. **d** The expression of mitophagy-related proteins in mitochondria and ubiquitinated proteins in sEVs were analyzed by western blot assay (*n* ≥ 5, one-way ANOVA, *p < 0.05, **p < 0.01). **e**, **f** In vitro lysosome function and mitophagy in *Cd38*^−*/−*^ VSMCs with or without *Sirt1* or *Sirt3* interference were analyzed. *Cd38*^−*/−*^ VSMCs were transfected overnight with siRNA or siRNA plus LAMP1-mGFP plasmids. Ang II (100 nM) was administered and maintained for 3 days. The lysosomal structures (**e**) and ubiquitinated proteins in sEVs (**f**) were detected by cellular microfluorescence imaging and western blot assay (*n* ≥ 5, one-way ANOVA, *p < 0.05, **p < 0.01)

Cell homeostasis is maintained by the functional connections between organelles. Defects in mitochondria or lysosomes are able to induce mutual impairments of functions, suggesting that a signal axis exists between mitochondria or lysosomes.^33^ Lysosomal-associated membrane protein 1 (LAMP1), which is highly enriched in late endosomes and lysosomes, is used as a marker for these organelles in cells. Our results showed that Ang II stimulation induced the enlargement of LAMP1-mGFP-positive (LAMP1-mGFP+) lysosomes in cells after transfection of the LAMP1-mGFP plasmid (Fig. 7a) and increased mitophagy-associated proteins such as PTEN-induced kinase 1 (PINK1), Parkin RBR E3 ubiquitin-protein ligase (Parkin) and sequestosome 1 (p62) in mitochondria, as well as ubiquitinated protein levels of sEVs, in VSMCs (Fig. 7b). These results suggested that Ang II initiated mitophagy to eliminate damaged mitochondria, whereas the increase in mitochondrial components and ubiquitinated proteins caused by impairments in lysosomal degradation function could be taken up by sEVs and ultimately released via SA-sEVs. Our results showed that the impaired lysosomes coupled with abnormal sEV secretion (increased ubiquitinated proteins) were reestablished upon CD38 deficiency (Fig. 7a, b), NAD supplementation (Fig. 7c, d), or the overexpression of *Sirt1* and *Sirt3* (Supplementary Fig. S8c, d) in WT VSMCs. In addition, the lysosome diameters (Fig. 7e) were further increased by siRNA-mediated knockdown of *Sirt1* or *Sirt3*, and the Ang II-induced expression levels of ubiquitinated proteins from sEVs (Fig. 7f) were further elevated in *Cd38*^*−/−*^ VSMCs, compared with those in the control group. All these results demonstrated that CD38 deficiency effectively restored lysosomal dysfunction and promoted mitophagy-mediated damaged mitochondrial degradation, indicating that CD38 deficiency-mediated regulation was dependent on intracellular NAD levels and SIRT1/SIRT3 activities.

### SA-sEVs were more easily internalized by senescent VSMCs

**Fig. 8:**
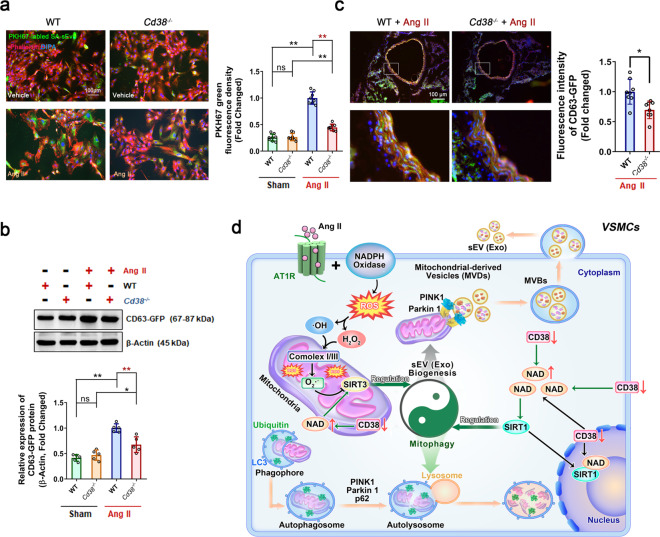
CD38 deficiency prevented the uptake of SA-sEVs by VSMCs after Ang II challenge. **a**, **b** In vitro sEV internalization was analyzed in VSMCs with Ang II or Vehicle stimulation. **(a)** Internalized SA-sEVs were detected by cellular microfluorescence imaging in VSMCs treated overnight with PKH-67 fluorescent dye-labeled SA-sEVs derived from senescent VSMCs (green dots represent sEVs, *n* ≥ 5, one-way ANOVA, *p < 0.05, **p < 0.01). **(b)** GFP protein expression in VSMCs treated overnight with SA-sEVs derived from CD63-GFP VSMCs was detected by western blot analysis. GFP protein expression represented the quantity of SA-sEV uptake by VSMCs (*n* ≥ 5, one-way ANOVA, *p < 0.05, **p < 0.01). **c** Ex vivo internalization was analyzed in vessels of WT and *Cd38*^*−/−*^ mice stimulated with Ang II. SA-sEVs, including CD63-GFP+ exosomes, were purified from senescent WT VSMCs. Then, SA-sEVs were incubated with aortas of WT and *Cd38*^*−/−*^ mice pretreated with Ang II (1 μM) for 24 h. SA-sEV internalization in aortas after overnight incubation was detected by immunofluorescent staining (*n* = 8, unpaired *t*-test, *p < 0.05, **p < 0.01). **d** CD38 deficiency-induced increases in intracellular NAD levels inhibited SA-sEV generation by reestablishing lysosome function and promoting mitophagy. Functional connections between cell organelles maintain cell homeostasis. The balance between mitophagy and mitochondria-derived MVB formation determines the fate of damaged mitochondria in senescent cells. Ang II stimulation promoted intracellular ROS production and induced mitochondrial dysfunction, which is critical for VSMC senescence in hypertension. During the aging process, injured mitochondria selectively enter the endolysosomal pathway to disrupt lysosome function and mitophagy and ultimately enhance MVB exosome biogenesis. SA-sEVs, including exosomes released from senescent VSMCs, are important SASP components that exacerbate the aging of neighboring cells. CD38 deficiency led to elevated SIRT1 and SIRT3 activities by increasing cellular NAD levels and reestablished mitochondrial and lysosomal functions to inhibit SA-sEV generation and SASP acquisition (sEVs small extracellular vesicles, Exo exosomes, SA-sEVs senescence-associated sEVs, MVBs multivesicular bodies, SASP senescence-associated secretory phenotype)

To further investigate the roles of SA-sEVs in promoting VSMC senescence, an uptake analysis of SA-sEVs was performed in vitro and in vivo. SA-sEVs from Ang II-challenged WT VSMCs were labeled with green fluorescent PKH67 dye and then incubated with VSMCs pretreated with Ang II for 3 days. The results showed that Ang II-treated senescent VSMCs were able to take up more SA-sEVs than vehicle-treated VSMCs; in contrast, CD38 deficiency inhibited SA-sEV internalization (Fig. 8a). The Western blot analysis also showed that CD38 deficiency led to alleviated Ang II-induced increases in GFP protein expression in senescent cells (Fig. 8b). In vivo experiments were performed in which thoracic aortas obtained from WT and *Cd38*^*−/−*^ mice were incubated with Ang II for one day to induce vascular senescence and then incubated with high levels of SA-sEVs secreted overnight from CD63-GFPVSMCs. The images showed that after Ang II stimulation, less SA-sEVs were internalized by the aortas from the *Cd38*^−*/−*^ mice compared to the WT aortas (Fig. 8c), suggesting that in the presence of CD38, the aging SA-sEVs were more easily and efficiently internalized by the Ang II-treated VSMCs in the aorta which possibly aggravated VSMC senescence.

## Discussion

In the present study, we made three important findings. First, we demonstrated that CD38 deficiency alleviated Ang II-induced hypertension and vascular remodeling by inhibiting VSMC senescence. The protection was verified by utilizing component 78c, a specific CD38 inhibitor. Ang II, as a major component of the renin–angiotensin–aldosterone system, which has been reported to mediate age-dependent cardiomyopathy and age-associated vasculopathy;^34^ accordingly, this regimen could effectively and repeatably trigger vascular/VSMC senescence in vivo and in vitro to mimic vascular aging in hypertension.^28,35^ NAD is a cofactor of multiple key enzymes involved in the tricarboxylic acid cycle, oxidative phosphorylation, glycolysis, and redox reactions. Moreover, NAD serves as a substrate for several enzymes, such as sirtuins and poly(ADP-ribose) polymerases (PARPs), which regulate numerous important cellular physiological and pathological processes. Increasing evidence indicates that NAD declines with aging, suggesting that maintaining intracellular NAD homeostasis is beneficial for halting senescence. Intracellular NAD homeostasis is dependent on NAD synthesis and consumption. CD38, a main NADase in mammalian cells, has been previously shown to be critical for NAD decreases with age,^11–13^ and its expression in nonsenescent cells has been reported to be driven by the senescence-associated secretory phenotype (SASP) effect.^36^ In previous studies, we observed that CD38 deficiency significantly alleviated various cardiovascular injuries, including myocardial ischemia–reperfusion injury^37,38^, and cardiac hypertrophy.^39^ In the current study, we demonstrated that CD38 not only contributed to vascular remodeling in hypertension but also played a detrimental pro-senescence role in VSMC senescence, further confirming the correlation of NAD decline and aging. Considering that CD38 exists on the surface of immune cells, particularly T lymphocytes, bone marrow chimeras were established to assess whether immune cells with normal CD38 expression can independently eliminate the protection against Ang II-induced hypertension and vascular remodeling in *Cd38*^*−/−*^ mice. Interestingly, the protective effect was sustained in the group of *Cd38*^*−/−*^ mice with WT bone marrow transplants, indicating that loss of CD38 in lymphocyte cells derived from bone marrow was not essential to prevent Ang II-induced vascular modeling.

Moreover, reversion of intracellular NAD levels and CD38 activity inhibition have been previously demonstrated to greatly improve aging-related metabolic dysfunctions, which correlate with glucose tolerance, muscle function, exercise capacity, and cardiac function.^29^ It has also been reported that daratumumab, an anti-CD38 antibody, has been utilized to treat multiple myeloma clinically successfully.^40^ Similarly, a potent and specific CD38 inhibitor (compound 78c) has been shown to ameliorate age-related metabolic dysfunction.^29^ In our present study, we also demonstrated that either supplementation with exogenous NAD or administration of the specific CD38 inhibitor compound 78c significantly ameliorated Ang II-induced VSMC senescence. Considering the excellent consistency of our results, our study suggested that CD38 may be a potential target for drug development for treating age-related diseases.

Second, we demonstrated that CD38 deficiency reduced VSMC senescence by suppressing the biogenesis, secretion, and internalization of senescence-associated sEVs (SA-sEVs). Exosomes (30–150 nm) and microvesicles (50–1000 nm) are generally recognized as highly heterogeneous extracellular vesicle populations that can be prepared with ultracentrifugation technology.^14^ Exosomes play important roles in communication between cells and organs. Intraluminal vesicles (ILVs), considered exosome precursors, are initially generated from endosomes and evolve into multivesicular bodies (MVBs). Under stimulation, MVBs undergo exocytic fusion with the plasma membrane followed by release into the extracellular space. HRS is essential for endosomal ubiquitinated protein sorting and MVB formation. In our study, we utilized the infusion protein HRS-RFP to track MVB formation, which partially represents exosome biogenesis. We demonstrated for the first time that Ang II promoted the production and secretion of sEVs from senescent cells, in which CD38 deficiency alleviated these Ang-II-induced alterations. In addition, more HRS-HFP+MVBs with enlarged shapes, which were restored by CD38 deletion or NAD supplementation, were observed in senescent cells, suggesting that CD38 enhanced sEV biogenesis and MVB formation.

Although sEVs were discovered decades ago, they were recently identified as component of the SASP effect in aging progression. It is also known that sEVs play similar roles as cytokines, chemokines, matrix proteases, and growth factors. Various cellular stresses, such as oxidative stress,^41^ irradiation^42^, and telomere attrition,^43^ initiate the biogenesis and secretion of sEVs. In our study, we observed that sEVs derived from Ang II-challenged smooth muscle cells accelerated the senescence of neighboring undamaged cells. Moreover, SA-sEVs were easily internalized by senescent cells, exacerbating the aging process in these cells. Accordingly, these results advance our comprehension of the mechanisms of VSMC senescence in hypertension and provide insight into novel therapeutic targets for reducing vascular aging. However, the components in sEVs that are critical for the effects of the senescence-associated secretory phenotype remain unknown and need to be investigated in the future.

Third, we further demonstrated that CD38 deficiency markedly inhibited the biogenesis of SA-sEVs by maintaining lysosomal function and promoting mitophagy in a NAD-dependent manner. Intracellular NAD levels are essential for the activation of sirtuin proteins, the expression of which greatly improves health and longevity. In our study, we found that VSMC senescence was aggravated upon SIRT1 or SIRT3 expression inhibition with specific siRNAs, suggesting that CD38 exacerbated VSMC senescence of VSMCs by suppressing SIRT1 and SIRT3 activities by reducing the intracellular NAD concentration. Additionally, we observed that exosomal protein CD63-GFP accumulation and HRS-RFP+MVB formation in VSMCs were enhanced upon knockdown of *Sirt1* and *Sirt3*, suggesting that SIRT1 and SIRT3 may participate in sEV-mediated smooth muscle cell senescence.

Mitochondria are important energy-producing organelles that constitute approximately one-fifth of the cell volume and generate >80% cellular ATP in VSMCs. A decline in mitochondrial quality and activity with aging has been previously correlated with the development of age-related diseases.^31^ Functional correlations between cellular organelles play key roles in maintaining homeostasis between mitochondria, lysosomes, and sEV biogenesis-associated endosomes. Emerging evidence indicates that lysosomal activity is impaired as a consequence or as the origin of deficient mitochondrial function. Previous studies have shown that gene ablation or pharmacological inhibition of mitochondrial proteins such as mitochondrial transcription factor A (TFAM), apoptosis-inducing factor mitochondria associated 1 (AIF), and mitochondrial dynamin, including GTPase OPA1 and PINK1, altered lysosome morphology and impaired mitochondrial activities, thereby resulting in disruption of endolysosomal trafficking and mitophagy.^44^ Furthermore, lysosomal alkalization-induced lysosomal dysfunction has been shown to lead to mitophagy impairment in pancreatic β cells,^45^ and abnormal secretome generation leading to excess exosome biogenesis has been found in breast cancer cells,^23^ suggesting that the deficient mitochondrial-lysosomal axis promotes mitochondria-endosome fusion and mitochondria-derived sEV generation. These phenomena have been recently reported to be involved in the pathogenesis of age-related neurodegenerative diseases, including Alzheimer’s disease,^46^ Parkinson’s disease,^47^ and Huntington disease.^48^ In addition, Ang II is able to cause mitochondrial injury by increasing oxidative stress. In our study, the lysosome enlargement and functional disorder induced by Ang II stimulation resulted in lysosome dysfunction and mitophagy impairment, suggesting that more mitochondrial compounds were sorted into exosomes and secreted through MVBs. Either *Cd38* gene deletion or NAD supplementation successfully reinstated the alterations caused by Ang II stimulation and inhibited sEV packaging of mitochondrial compounds and ubiquitinated proteins, suggesting that CD38 deficiency promoted lysosome-mediated degradation. CD38 deficiency-mediated protection was reversed by SIRT1 or SIRT3 inhibition, especially SIRT3 inhibition, demonstrating that mitophagy facilitated damaged mitochondrial elimination. Taken together, the findings from our study demonstrated that CD38 deficiency inhibited the biogenesis and secretion of sEVs by maintaining mitochondrial-lysosomal function and promoting mitophagy in a NAD-SIRT1/SIRT3-dependent manner (Fig. 8d).

In conclusion, our study demonstrated that CD38 deficiency significantly alleviated Ang II-induced hypertension and vascular remodeling by inhibiting the SA-sEV-mediated senescence of VSMCs. Moreover, CD38 deficiency-mediated inhibition of the biogenesis, secretion, and internalization of SA-sEVs by senescent VSMCs was partially involved in reestablishing the function of the mitochondrial-lysosomal axis in smooth muscle cells. Finally, our results provide strong evidence that CD38 inhibition or NAD supplementation may serve as potential therapeutic strategies for age-related diseases.

## Materials and methods

### Animals

All study experiments adhered to the National Institutes of Health Guidelines on the Use of Laboratory Animals and were approved by the *Sichuan University Committee on Animal Care*. Eight- to ten-week-old male *Cd38*^*−/−*^ mice and age and genetically matched wild-type mice (WT, C57BL/6) were randomly assigned to the sham or Ang II-treatment group and were infused with saline or Ang II (490 ng/min/kg via subcutaneously implanted osmotic pumps for 4 weeks), respectively. CD38 inhibitor (78c) and NMN were administered to WT mice by intraperitoneal injection (i.p., 10 mg/kg/dose) twice daily^29^ and by oral gavage (300 mg/kg) daily^49^ over a period of 4 weeks along with Ang II infusion.

### Bone marrow transplantation assay

Bone marrow chimeras were produced as previously reported.^25,50^ The recipient mice were irradiated with a split dose of 800 Rads, and subsequently, 2 × 10^6^ bone marrow cells from donor mice were injected retro-orbitally into irradiated recipient mice. At least 6 weeks after irradiation and reconstitution, the recipient animals were used in subsequent Ang II infusion experiments, and the expression of CD38 in peripheral blood T lymphocytes was confirmed by flow cytometry.

### Blood pressure detection

The noninvasive tail-cuff method was used to monitor the caudal arterial pressure weekly and at the endpoint of 4 weeks of ANG II infusion. Systolic blood pressure (SBP) and diastolic blood pressure (DBP) were measured by the intra-arterial catheter method through the left common carotid artery under anesthetization with isoflurane (2%) in oxygen using an eight-channel physiological recorder (iWorx 308, iWorx/CB Sciences, Dover, NH, USA).

### Mouse VSMC culture and selection of stable CD63-GFP/COX8-GFP VSMCs

Mouse VSMCs were isolated and cultured using a standard enzymatic digestion technique. The aorta was excised from male *Cd38*^49^ mice and age and genetically matched wild-type mice. The vessels were cleaned of adipose and connective tissue and placed in an enzymatic solution (1.42 mg/ml collagenase II) for 4–6 h. The cells were cultured in DMEM with 10% FBS and penicillin/streptomycin (100 U/ml). Stable CD63-GFP VSMCs and COX8-GFP VSMCs were generated by transfection with lentiviruses pCT-CD63-GFP (System Biosciences, CYTO120-VA-1) and pCT-Mito-GFP (System Biosciences, CYTO102-VA-1) for at least 6 h, recovered for 24 h in fresh medium, and subjected to selection with puromycin (2 μg/ml, Sigma, P9620) for 48 h. Hence, the sEVs generated in the cells were labeled with GFP, which made it easy to track the sEV signal under a fluorescence microscope.

### SA-β-gal staining

SA-β-gal activity was determined using a senescence β-galactosidase staining kit (CST, #9860). Briefly, VSMCs were plated at low density (5 × 10^4^) in 12-well plates. After incubating in DMEM containing 1% FBS overnight to induce synchronization, the VSMCs were cultured with Ang II for 3 days. Fresh NAD or NAM stimulants/inhibitors were added every day during the 3-day incubation period. Then, the cells were washed and fixed with 1× fixative solution for 10–15 min at room temperature. After washing twice with PBS, the cells were incubated with β-galactosidase staining solution and incubated at 37 °C at least overnight (~16–24 h). The cells were washed twice with PBS and maintained in 70% glycerol. SA-β-gal images were randomly obtained using a light microscope (Olympus, Japan). The percentage of stained senescent cells was determined with ImageJ software (NIH, Bethesda, MD, USA) using at least five visual fields of each group.

### Western blot analysis

Proteins were extracted from tissues or cells using cell lysis buffer (10×) (CST, 9803) supplemented with a protease inhibitor cocktail (Thermo Fisher Scientific, 78438). Fifty micrograms of protein were separated by SDS-PAGE and transferred onto a PVDF membrane, which was blocked with 5% skim milk. The membranes were incubated with primary antibodies overnight at 4 °C and then incubated with secondary HRP-conjugated antibody at room temperature for 2 h. The bands were visualized and analyzed using an enhanced chemiluminescent (ECL) detection system (Image Lab, Bio-Rad). β-Actin and VDAC were used as loading controls. The primary antibodies were anti-phospho-histone H2A.X (Ser139) (20E3) anti-rabbit mAb (CST, #9718), anti-histone H2A.X antibody (CST, #2595), anti-p21/CIP1/CDKN1A antibody (NOVUS, NB100-1941), anti-CDKN2A/p16INK4a antibody (Abcam, ab211542), anti-TSG101 antibody (Proteintech, 14497-1-AP), anti-CD63 antibody [EPR21151] (Abcam, ab217345), purified anti-mouse/rat CD81 antibody (BioLegend, 104901), anti-Flotillin-1 (D2V7J) XP^®^ rabbit mAb (CST, #18634), anti-SIRT1 rabbit polyclonal antibody (Proteintech, 13161-1-AP), anti-SIRT3 rabbit polyclonal antibody (Proteintech, 10099-1-AP), anti-PINK1 (D8G3) Rabbit mAb (CST, #6946), anti-Parkin (Prk8) mouse mAb (CST, #4211); anti-SQSTM1/p62 (D1Q5S) rabbit mAb (CST, #39749), anti-ubiquitin Antibody (CST, #3933), anti-VDAC (D73D12) Rabbit mAb (CST, #4661), and anti-β-actin antibody (C4) (Santa Cruz, sc-47778).

### sEV purification and identification

Primary VSMC culture medium was centrifuged at 2000 × *g* for 30 min and then at 10,000 × *g* for 40 min to remove dead cells and cell debris. Then, the supernatant sera and culture media were filtered through 0.22-μm filters and ultracentrifuged at 100,000×*g* for 1–2 h at 4 °C. Pelleted sEVs were suspended in PBS, ultracentrifuged again at 100,000×*g* for washing, resuspended in PBS, and prepared for subsequent studies. The characterization as sEVs was confirmed by measuring the expression of the exosome-specific markers CD63, CD81, and TSG101 via western blot analysis. The particle sizes and numbers were measured by NanoSight analysis (NS300, Malvern Instruments) and transmission electron microscopy (TEM, H-600, Hitachi, Japan).

### sEV trafficking detection

To monitor sEV trafficking in vitro, sEVs were labeled with PKH67 fluorescent dye using a PKH67 fluorescent cell linker kit (Sigma-Aldrich) following the manufacturer’s protocol. PKH67-labeled sEVs were incubated with VSMCs for 12 h, fixed with 4% paraformaldehyde for 10 min at room temperature, and washed twice with PBS. After incubation with 1% BSA for 20-30 min, the cells were stained with 5 U/ml rhodamine-phalloidin (ThermoFisher Scientific, R415) for 20 min and 1 μg/ml DAPI (4′,6-diamidino-2′-phenylindole, dihydrochloride, Sigma, D9542) for 5 min at room temperature.

To monitor sEV trafficking ex vitro, sEVs carrying CD63-GFP purified from the culture medium of the stable CD63-GFP VSMC cell line were used. The sEVs were incubated with aortas overnight. sEV internalization was detected by immunofluorescent staining. The aortas or mesentery arteries were fixed overnight in 4% paraformaldehyde, embedded in optimal cutting temperature compound (OCT), cut into 10-μm-thick sections, and mounted on glass slides for staining. The tissues were fixed in 2% formaldehyde in PBS for 20 min and washed with PBS three times. Then, the sections were covered with ice-cold 100% methanol for 10 min at −20 °C, washed three times, and blocked with 0.5% casein in PBS buffer for 1 h at room temperature. Tissue F-actin was stained with 5 U/ml rhodamine-phalloidin. Nuclei were stained with DAPI (Vector Laboratories, H-1200). Micrographs of all immunostained tissues were acquired via an Olympus BX51 fluorescence microscope and Olympus DP72 and DP74 cameras (Japan).

### Histology and morphometric analysis

Thoracic aortas and mesenteric arteries were isolated and processed for paraffin embedding and then cut into 4-μm sections. Sections from each cluster were stained with hematoxylin–eosin (H&E), Masson, Orcein, and Gomori’s aldehyde-fuchsin dye to detect the structure, collagen deposition, and elastin content. The luminal radius, media thickness, and intensity of collagen and elastin staining were measured by ImageJ software, and the media-to-lumen ratio was calculated. In brief, the thoracic aorta thickness was determined by measuring the distance from the internal elastic lamina (IEL) to the external elastic lamina (EEL). For each slide, 4 points (at the 12, 3, 6, and 9 o’clock positions) were measured and averaged. The media area was determined by measuring the area between the IEL and EEL. Then, the media-to-lumen ratio was calculated. The thoracic aorta paraffin slides were deparaffinized and subjected to antigen retrieval in hot citric acid (H-3300, Vector Laboratories, CA94010). After cooling, the slides were permeabilized with 0.2% Triton-100 for 15 min, blocked with 1% BSA or 0.5% casein in PBS for 2 h at room temperature, and incubated overnight with anti-CD38 antibody [EPR21079] (Abcam, ab216343), phospho-histone H2A.X (Ser139) (20E3) Rabbit mAb (CST, #9718), p21/CIP1/CDKN1A antibody (NOVUS, NB100-1941) and anti-CDKN2A/p16INK4a antibody (Abcam, ab211542) at 4 °C. After washing, a diluted secondary antibody was added and incubated for 1 h at room temperature. Then, DAB substrate was added for coloration. For immunofluorescence staining, a secondary antibody (donkey anti-rabbit IgG (H + L) highly cross-adsorbed secondary antibody, Alexa Fluor 594) was used. Micrographs of all immunostained tissues were acquired via an Olympus DP74 camera (Japan). In addition, the ratio of p-H2A.X^ser139^-, p21^Waf1/Cip1^- and p16^INK4A^-positive cells and the intensity of the CD38 staining was measured by ImageJ software. The intensity of the fluorescence is equal to the total optical density of fluorescence in the image divided by the total area of fluorescence. The calculations for immunohistochemical staining were similar.

For cell immunofluorescence staining, the cells were cultured on Millicell® EZ SLIDES (Millipore, C86024). After treatment, the cells were fixed with 4% paraformaldehyde for 10 min at room temperature and washed with PBS three times. Then, the slides were covered with ice-cold 100% methanol for 10 min at −20 °C, washed three times, and blocked with 0.5% casein PBS buffer for 1 h at room temperature. Then, the slides were incubated overnight with primary antibody at 4 °C and secondary antibody (donkey anti-rabbit IgG (H + L) highly cross-adsorbed secondary antibody, Alexa Fluor 594) for 1 h at room temperature. Finally, a mounting medium with DAPI-Aqueous Fluoroshield (Abcam, ab104139) was added. After image acquisition, the red fluorescent dots representing DNA damage (p-H2A.X^ser139^) in every nucleus, and the p21^Waf1/Cip1^- and p16^INK4A^-positive nuclei were measured by ImageJ software.

### Transfection of siRNAs and plasmids

siRNAs targeting *Sirt1* and *Sirt3* and their corresponding negative controls (NC) were synthesized by Invitrogen; Thermo Fisher Scientific, Inc. siRNA was mixed with HiPerFect transfection reagent (Qiagen, 301705) and administered to cells for 6 h per the manufacturer’s protocol. Attractene transfection reagent (Qiagen, 301005) was used in plasmid overexpression experiments. Then, the medium was changed to a complete culture medium without antibiotics. After overnight siRNA or plasmid transfection, the cells were challenged with Ang II for 3 days.

### Statistical analysis

The data are reported as the means ± SD. For analysis of the differences between the two groups, an unpaired Student’s *t*-test was performed. For multiple groups, the data were analyzed by one-way (ANOVA) followed by Tukey’s and Dunnett’s multiple comparison tests (details in figure legends). For all statistical tests, *p* values < 0.05 were considered statistically significant. All statistical analyses were performed with GraphPad Prism 8.

## Supplementary information

Revised Supplementary Material

## Data Availability

All data that support the findings of this study are available from the corresponding authors upon reasonable request.

## References

[1] Childs BG, Durik M, Baker DJ, van Deursen JM (2015). Cellular senescence in aging and age-related disease: from mechanisms to therapy. Nat. Med..

[2] van Deursen JM (2014). The role of senescent cells in ageing. Nature.

[3] Sano M (2007). p53-induced inhibition of Hif-1 causes cardiac dysfunction during pressure overload. Nature.

[4] Childs BG (2016). Senescent intimal foam cells are deleterious at all stages of atherosclerosis. Science.

[5] Chi C (2019). Vascular smooth muscle cell senescence and age-related diseases: state of the art. Biochim. Biophys. Acta Mol. Basis Dis..

[6] Westhoff JH (2008). Hypertension induces somatic cellular senescence in rats and humans by induction of cell cycle inhibitor p16INK4a. Hypertension.

[7] Noureddine H (2011). Pulmonary artery smooth muscle cell senescence is a pathogenic mechanism for pulmonary hypertension in chronic lung disease. Circ. Res..

[8] Gomes AP (2013). Declining NAD(+) induces a pseudohypoxic state disrupting nuclear-mitochondrial communication during aging. Cell.

[9] Mouchiroud L (2013). The NAD(+)/Sirtuin pathway modulates longevity through activation of mitochondrial UPR and FOXO signaling. Cell.

[10] Hogan KA, Chini CCS, Chini EN (2019). The multi-faceted ecto-enzyme CD38: roles in immunomodulation, cancer, aging, and metabolic diseases. Front. Immunol..

[11] Camacho-Pereira J (2016). CD38 dictates age-related NAD decline and mitochondrial dysfunction through an SIRT3-dependent mechanism. Cell Metab..

[12] Chini CCS (2020). CD38 ecto-enzyme in immune cells is induced during aging and regulates NAD(+) and NMN levels. Nat. Metab..

[13] Covarrubias AJ (2020). Senescent cells promote tissue NAD(+) decline during ageing via the activation of CD38(+) macrophages. Nat. Metab..

[14] Sluijter JPG (2018). Extracellular vesicles in diagnostics and therapy of the ischaemic heart: Position Paper from the Working Group on Cellular Biology of the Heart of the European Society of Cardiology. Cardiovasc. Res..

[15] Gan L (2020). Small extracellular microvesicles mediated pathological communications between dysfunctional adipocytes and cardiomyocytes as a novel mechanism exacerbating ischemia/reperfusion injury in diabetic mice. Circulation.

[16] Jakhar, R. & Crasta, K. Exosomes as emerging pro-tumorigenic mediators of the senescence-associated secretory phenotype. *Int. J. Mol. Sci.* **20**, 2547 (2019). 10.3390/ijms20102547 PMC6566274 31137607

[17] Terlecki-Zaniewicz L (2018). Small extracellular vesicles and their miRNA cargo are anti-apoptotic members of the senescence-associated secretory phenotype. Aging.

[18] Kadota T (2018). Emerging role of extracellular vesicles as a senescence-associated secretory phenotype: insights into the pathophysiology of lung diseases. Mol. Asp. Med..

[19] Yang J, Yu XF, Li YY, Xue FT, Zhang S (2019). Decreased HSP70 expression on serum exosomes contributes to cardiac fibrosis during senescence. Eur. Rev. Med. Pharm. Sci..

[20] Fulzele S (2019). Muscle-derived miR-34a increases with age in circulating extracellular vesicles and induces senescence of bone marrow stem cells. Aging.

[21] Hurwitz SN (2018). An optimized method for enrichment of whole brain-derived extracellular vesicles reveals insight into neurodegenerative processes in a mouse model of Alzheimer’s disease. J. Neurosci. Methods.

[22] Prattichizzo F (2017). Exosome-based immunomodulation during aging: a nano-perspective on inflamm-aging. Mech. Ageing Dev..

[23] Latifkar A (2019). Loss of Sirtuin 1 alters the secretome of breast cancer cells by impairing lysosomal integrity. Dev. Cell.

[24] Guedes AG (2015). CD38 and airway hyper-responsiveness: studies on human airway smooth muscle cells and mouse models. Can. J. Physiol. Pharm..

[25] Guedes AG (2015). Airway responsiveness in CD38-deficient mice in allergic airway disease: studies with bone marrow chimeras. Am. J. Physiol. Lung Cell Mol. Physiol..

[26] Bernardes de Jesus B, Blasco MA (2012). Assessing cell and organ senescence biomarkers. Circ. Res..

[27] Gorgoulis V (2019). Cellular senescence: defining a path forward. Cell.

[28] Li DJ (2016). alpha7 nicotinic acetylcholine receptor relieves angiotensin II-induced senescence in vascular smooth muscle cells by raising nicotinamide adenine dinucleotide-dependent SIRT1 activity. Arterioscler. Thromb. Vasc. Biol..

[29] Tarrago MG (2018). A potent and specific CD38 inhibitor ameliorates age-related metabolic dysfunction by reversing tissue NAD(+) decline. Cell Metab..

[30] Urbanelli, L., Buratta, S., Sagini, K., Tancini, B. & Emiliani, C. Extracellular vesicles as new players in cellular senescence. *Int. J. Mol. Sci.* **17**, 1408 (2016). 10.3390/ijms17091408 PMC5037688 27571072

[31] Sun N, Youle RJ, Finkel T (2016). The mitochondrial basis of aging. Mol. Cell.

[32] Yuan Y (2016). Regulation of SIRT1 in aging: roles in mitochondrial function and biogenesis. Mech. Ageing Dev..

[33] Picca A (2019). Mitochondrial dysfunction and aging: insights from the analysis of extracellular vesicles. Int. J. Mol. Sci..

[34] Yin H, Pickering JG (2016). Cellular senescence and vascular disease: novel routes to better understanding and therapy. Can. J. Cardiol..

[35] Kunieda T (2006). Angiotensin II induces premature senescence of vascular smooth muscle cells and accelerates the development of atherosclerosis via a p21-dependent pathway. Circulation.

[36] Chini C (2019). The NADase CD38 is induced by factors secreted from senescent cells providing a potential link between senescence and age-related cellular NAD(+) decline. Biochem. Biophys. Res. Commun..

[37] Guan XH (2016). CD38 deficiency protects the heart from ischemia/reperfusion injury through activating SIRT1/FOXOs-mediated antioxidative stress pathway. Oxid. Med. Cell Longev..

[38] Boslett J, Helal M, Chini E, Zweier JL (2018). Genetic deletion of CD38 confers post-ischemic myocardial protection through preserved pyridine nucleotides. J. Mol. Cell Cardiol..

[39] Guan XH (2017). CD38 promotes angiotensin II-induced cardiac hypertrophy. J. Cell Mol. Med..

[40] van der Veer MS (2011). The therapeutic human CD38 antibody daratumumab improves the anti-myeloma effect of newly emerging multi-drug therapies. Blood Cancer J..

[41] Atienzar-Aroca S (2016). Oxidative stress in retinal pigment epithelium cells increases exosome secretion and promotes angiogenesis in endothelial cells. J. Cell Mol. Med..

[42] Lehmann BD (2008). Senescence-associated exosome release from human prostate cancer cells. Cancer Res..

[43] Takasugi M (2017). Small extracellular vesicles secreted from senescent cells promote cancer cell proliferation through EphA2. Nat. Commun..

[44] Baixauli F (2015). Mitochondrial respiration controls lysosomal function during inflammatory T cell responses. Cell Metab..

[45] Assali EA (2019). Nanoparticle-mediated lysosomal reacidification restores mitochondrial turnover and function in beta cells under lipotoxicity. FASEB J..

[46] Xiao T (2017). The role of exosomes in the pathogenesis of Alzheimer’ disease. Transl. Neurodegener..

[47] Tofaris GK (2017). A critical assessment of exosomes in the pathogenesis and stratification of Parkinson’s disease. J. Parkinsons Dis..

[48] Jeon I (2016). Human-to-mouse prion-like propagation of mutant huntingtin protein. Acta Neuropathol..

[49] Mills KF (2016). Long-term administration of nicotinamide mononucleotide mitigates age-associated physiological decline in mice. Cell Metab..

[50] Misra RS (2010). G alpha q-containing G proteins regulate B cell selection and survival and are required to prevent B cell-dependent autoimmunity. J. Exp. Med..

