# Supplementary material for: CD38 deficiency alleviates Ang II-induced vascular remodeling by inhibiting small extracellular vesicle-mediated vascular smooth muscle cell senescence in mice
Source: Signal Transduct Target Ther. 2021 Jun 11;6:223. doi: 10.1038/s41392-021-00625-0 (PMC8192533; doi:10.1038/s41392-021-00625-0)
Supplement: Supplementary file 1 — Revised Supplementary Material [file 41392_2021_625_MOESM1_ESM.docx]

Supplementary Materials for

**CD38 deficiency alleviates Ang II-induced vascular remodeling by inhibiting small extracellular vesicle-mediated vascular smooth muscle cell senescence in mice**

Lu Gan^1,*^, Demin Liu^2^, Jing Liu^3^, Erya Chen^4^, Chan Chen^4^, Lian Liu^1^, Hang Hu^1^, Xiao-Hui Guan^5^, Wen Ma^1^, Yan-Zi Zhang^1^, Ya-Rong He^1^, Bo-Fu Liu^1^, Song-Ling Tang^1^, Wei Jiang^6^, Jian-Xin Xue^7,6,*^, Hong-Bo Xin^5,*^

**Correspondence to:** ganlu@wchscu.cn; killercell@163.com; [xinhb@ncu.edu.cn](mailto:xinhb@ncu.edu.cn)

This file includes:

Figures. S1 to S8

**
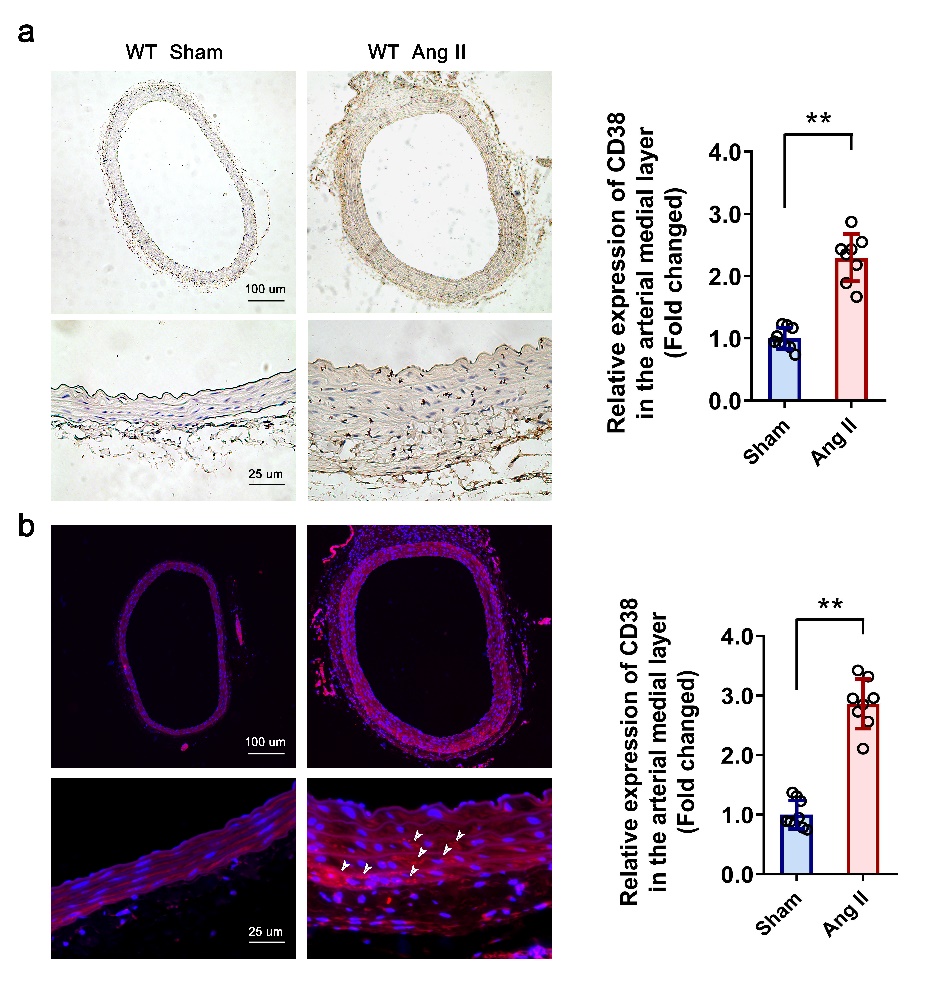
**

**Figure S1. The CD38 expression in aorta tissue after Ang II infusion.** After 4 weeks Ang II infusion, the CD38 expression in aorta were detected by (a) immunohistochemistry and (b) immunofluorescent staining. The white arrows indicated the accumulation of CD38 in VSMCs of the arterial medial layer after Ang II administration (n≥8, unpaired *t*-test, **p*<0.05, ***p*<0.01).

**
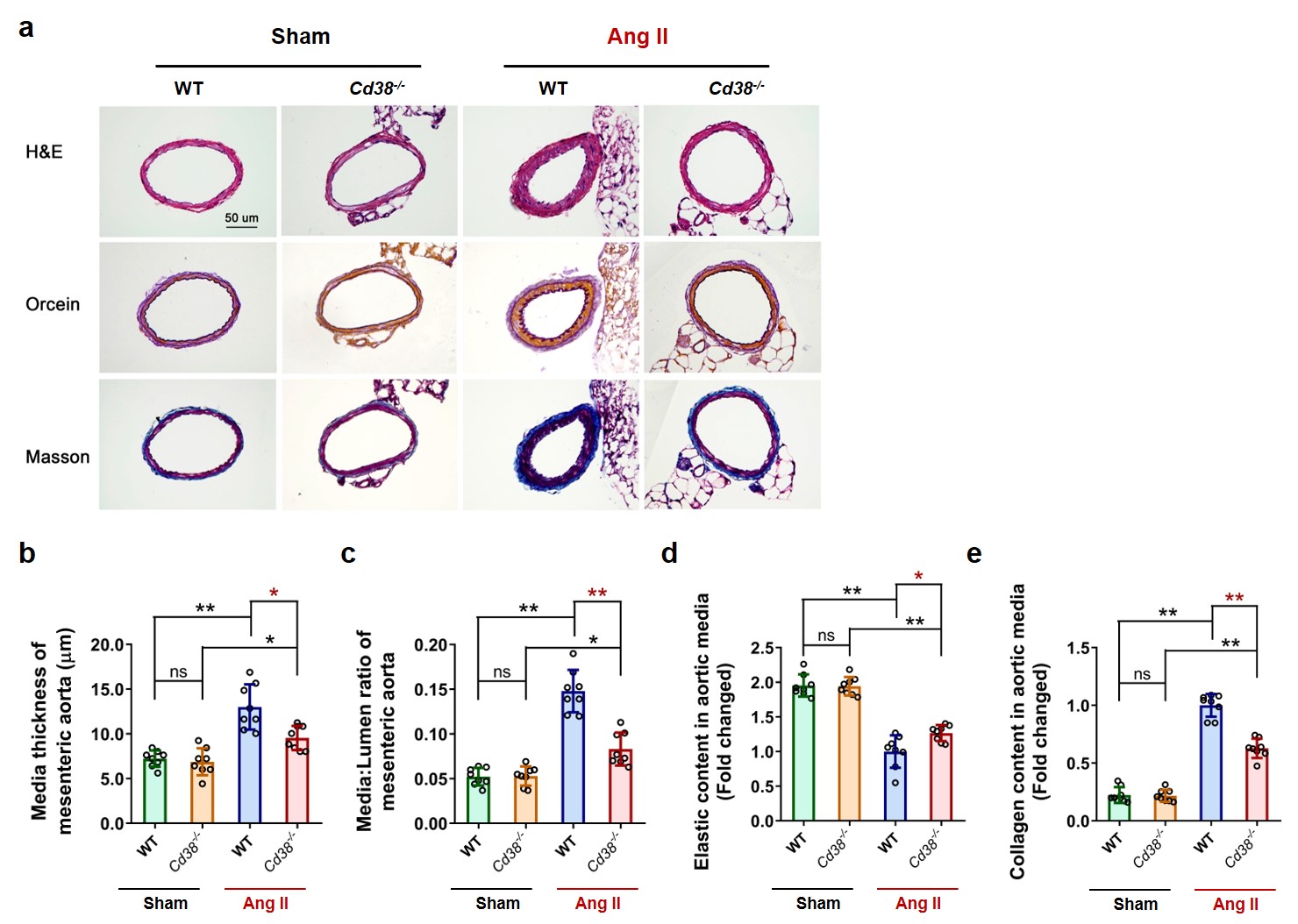
**

**Figure S2. CD38 deficiency alleviated Ang II infusion-induced remodeling of** **mesenteric artery.** (**a**) Vascular remodeling was analyzed in mesenteric artery sections obtained from WT and *Cd38^-/-^* mice with or without Ang II stimulation. Representative images of vessel sections stained with H&E, Orcein staining (dark reddish brown represented elastin) and Masson trichrome blue staining (blue represented collagen deposition). (**b-d**) media thickness (b), Media:Lumen ratio (c) of mesenteric arteries were calculated on the basis of the H&E staining, and the density of elastin (d) and collagen (e) in the mesenteric artery smooth muscle wall were quantitatively analyzed by Orcein staining and Masson trichrome blue staining, respectively (n=8, One-Way ANOVA, **p*<0.05, ***p*<0.01).

**
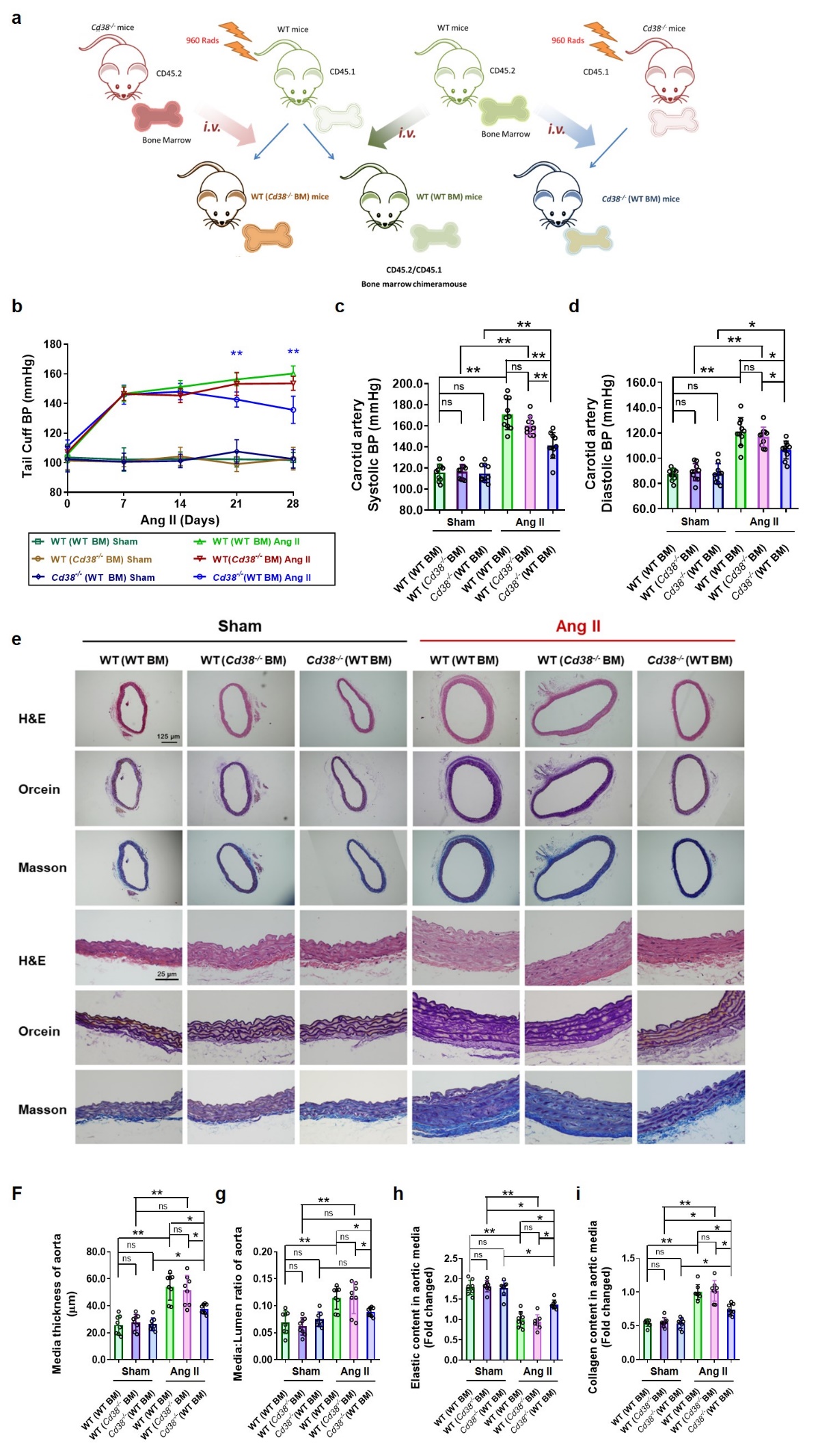
**

**Figure S3. The effects of lymphocytes on Ang II-induced hypertension and vascular remodeling by bone marrow transplantation in *Cd38*^-/-^ mice.** (**a**) The schematic diagram represented the bone marrow transplantation methods and the bone marrow chimera construction. *Cd38^-/-^ (WT BM)* mean that the bone marrow was replaced with WT donor cells in *Cd38^-/-^* recipient mice. WT *(Cd38^-/-^ BM)* mean that the bone marrow was replaced with *Cd38^-/-^* donor cells in WT recipient mice. (**b**) The caudal artery blood pressures were decreased in *Cd38^-/-^ (WT BM)* mice compared to *WT (WT BM)* mice with Ang II infusion (n≥10, Two-Way ANOVA, **p*<0.05, ***p*<0.01). (**c-d**) The systolic (c) and diastolic (d) blood pressures of carotid artery were restored in *Cd38^-/-^ (WT BM)* mice compared to *WT (WT BM)* mice with Ang II infusion. (n≥10, One-Way ANOVA, **p*<0.05, ***p*<0.01). (**e**) Vascular remodeling was analyzed in thoracic aorta sections from *WT (WT BM)*, WT (*Cd38^-/-^* BM) and *Cd38^-/-^ (WT BM)* mice with or without Ang II infusion. Representative images of vessel sections stained with H&E, Orcein staining (dark reddish brown/purple represented elastin) and Masson trichrome blue staining (blue represented collagen deposition). (**f**-**i**) Vascular remodeling analysis. Media thickness (f), Media:Lumen ratio (g) of aortas were calculated on the basis of H&E staining, and the density of elastin (h) and collagen (i) in aortic smooth muscle walls were quantitatively analyzed by Orcein staining and Masson trichrome blue staining, respectively. (n=8, One-Way ANOVA, **p*<0.05, ***p*<0.01).


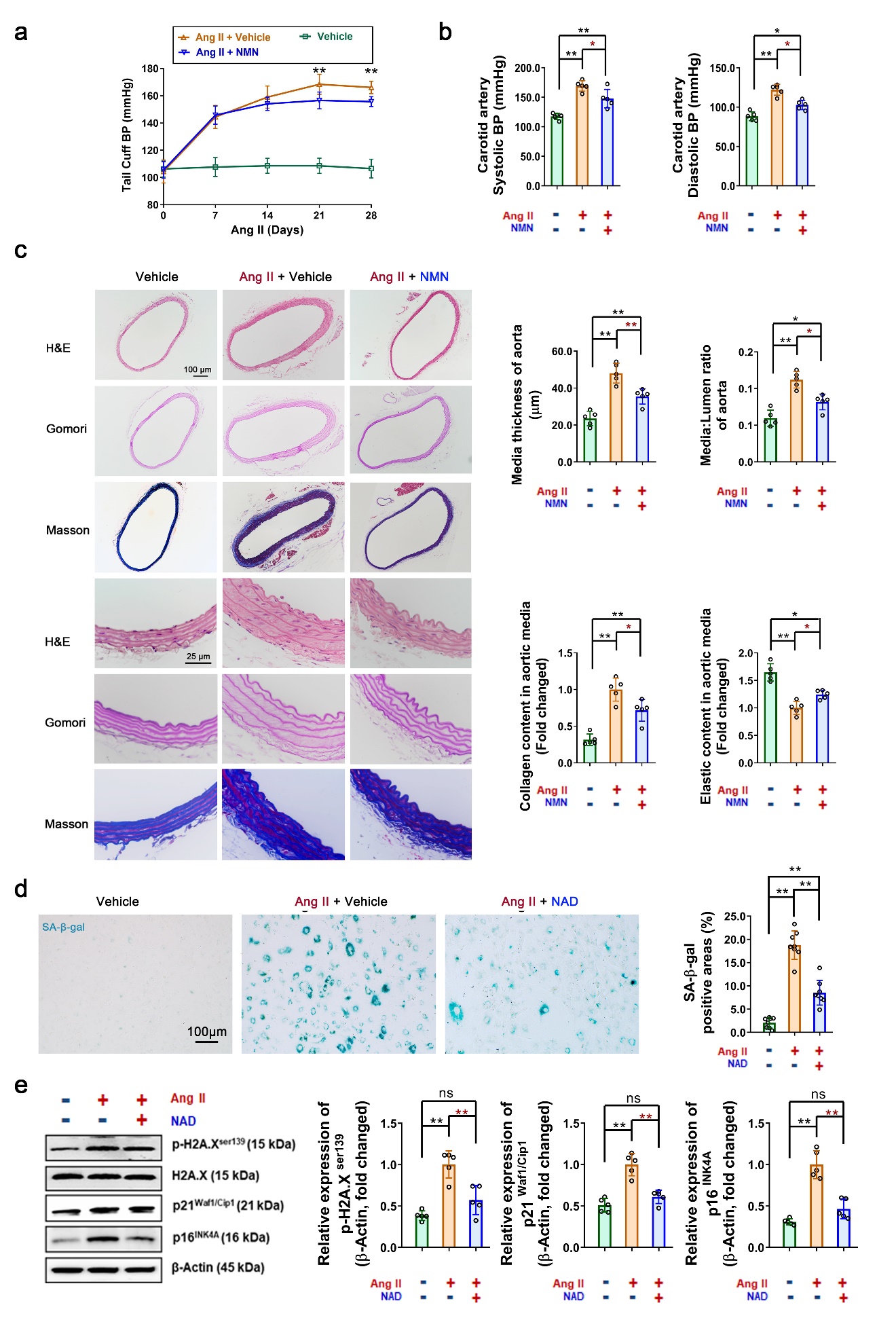


**Figure S4. NAD supplementation alleviated Ang II-induced hypertension, vascular remodeling and VSMC senescence.** (**a**) Caudal artery blood pressures were detected in WT mice with or without NMN administration every 7 days post Ang II infusion (n=5, Two-Way ANOVA, **p*<0.05, ***p*<0.01). (**b**) The systolic and diastolic blood pressures of carotid artery were detected in WT mice with or without NMN administration 4 weeks after Ang II infusion. (n=5, One-Way ANOVA, **p*<0.05, ***p*<0.01). (**c**) Vascular remodeling was analyzed in thoracic aorta sections obtained from WT mice with or without Ang II + NMN treatment. Representative images of vessel sections were stained with H&E, Gomori’s aldehyde-fuchsin staining (bright purple represented elastin) and Masson trichrome blue staining (blue represented collagen deposition). Media thickness, Media:Lumen ratio of aortas were calculated on the basis of H&E staining, and the density of elastin and collagen staining in aortic smooth muscle wall were quantitatively analyzed by Gomori’s aldehyde-fuchsin staining and Masson trichrome blue staining, respectively (n=5, One-Way ANOVA, **p*<0.05, ***p*<0.01). (**d-e**) NAD supplementation restored Ang II-induced cell senescence in *vitro*. NAD (100 μM) was added into the medium of VSMCs for 4 hours prior to Ang II administration. All these compounds were maintained in the medium for 3 days. And then the VSMCs were stained by (c) SA-β-gal and (d) the proteins were analyzed by western blot analysis (n≥5, One-Way ANOVA, **p*<0.05, ***p*<0.01).

**
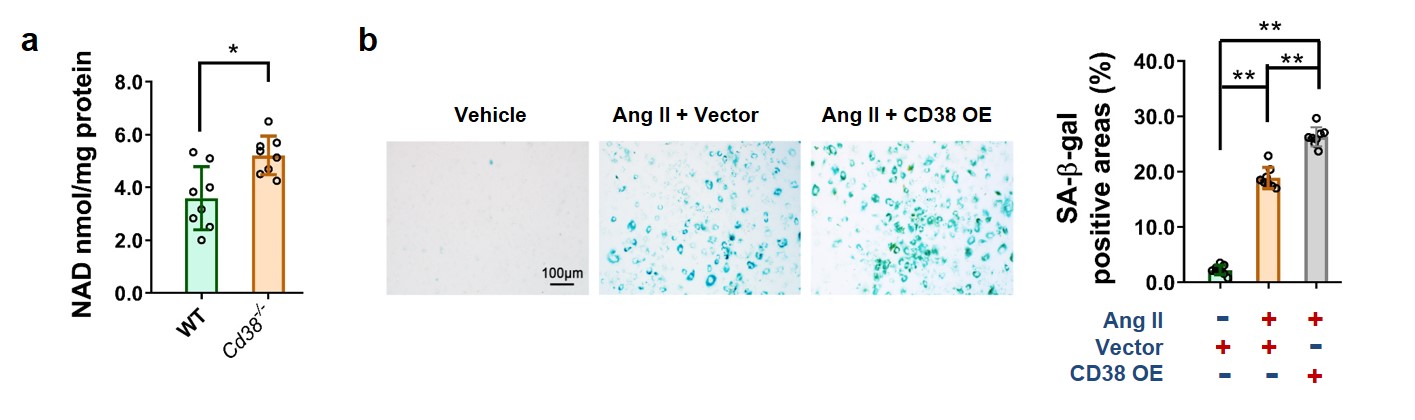
**

**Figure S5. Effects of CD38 overexpression on Ang II-induced VSMCs senescence.** (**a**) CD38 deficiency resulted in elevation of intracellular NAD levels. Total aorta NAD levels were measured by cycling assay in WT and *Cd38^-/-^* mice (n=8, unpaired t test, **p*<0.05, ***p*<0.01). (**b**) The VSMCs were transfected with plasmid overexpressing *Cd38* and NC plasmid. Overnight after transfection, Ang II were added and maintained for 3 days. And then VSMC senescence was analyzed by SA-β-gal staining. (n≥5, One-Way ANOVA, **p*<0.05, ***p*<0.01)

**
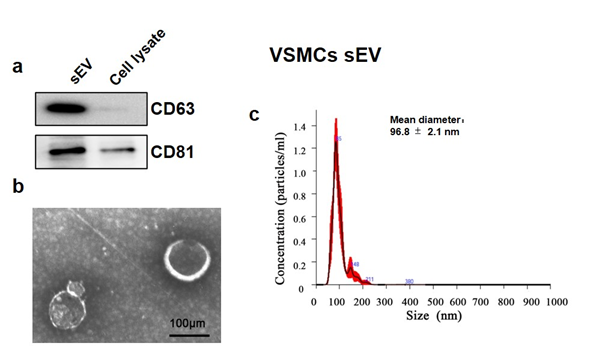
**

**Figure S6. Characteristics of VSMCs small extracellular vesicles (sEVs).** The VSMC sEVs were purified by ultracentrifugation methods. (**a**) The exosome markers CD63 and CD81 were detected by Western blot analysis. (**b**) The morphology of sEVs was characterized by transmission electron microscope (TEM). (**c**) Particle size distribution was determined by NanoSight tracking analysis.


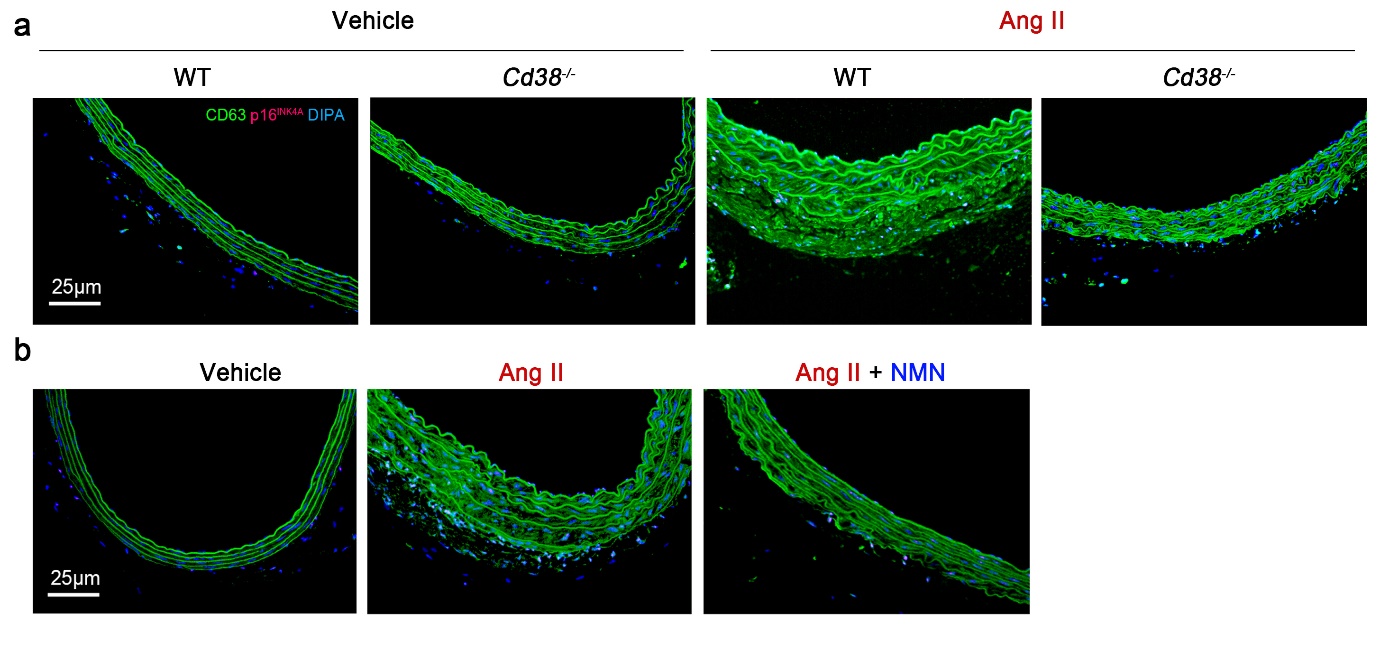


**Figure S7. CD63 expression in** **p16^INK4A^ positive VSMCs.** Four weeks After Ang II infusion in WT or *Cd38^-/-^* mice, or Four weeks After Ang II infusion plus NAM administration in WT mice, the CD63 (Green) combined with p16^INK4A^ (Red) expression in aorta were detected by immunofluorescent staining. The nucleus was stained by DIPA.

**
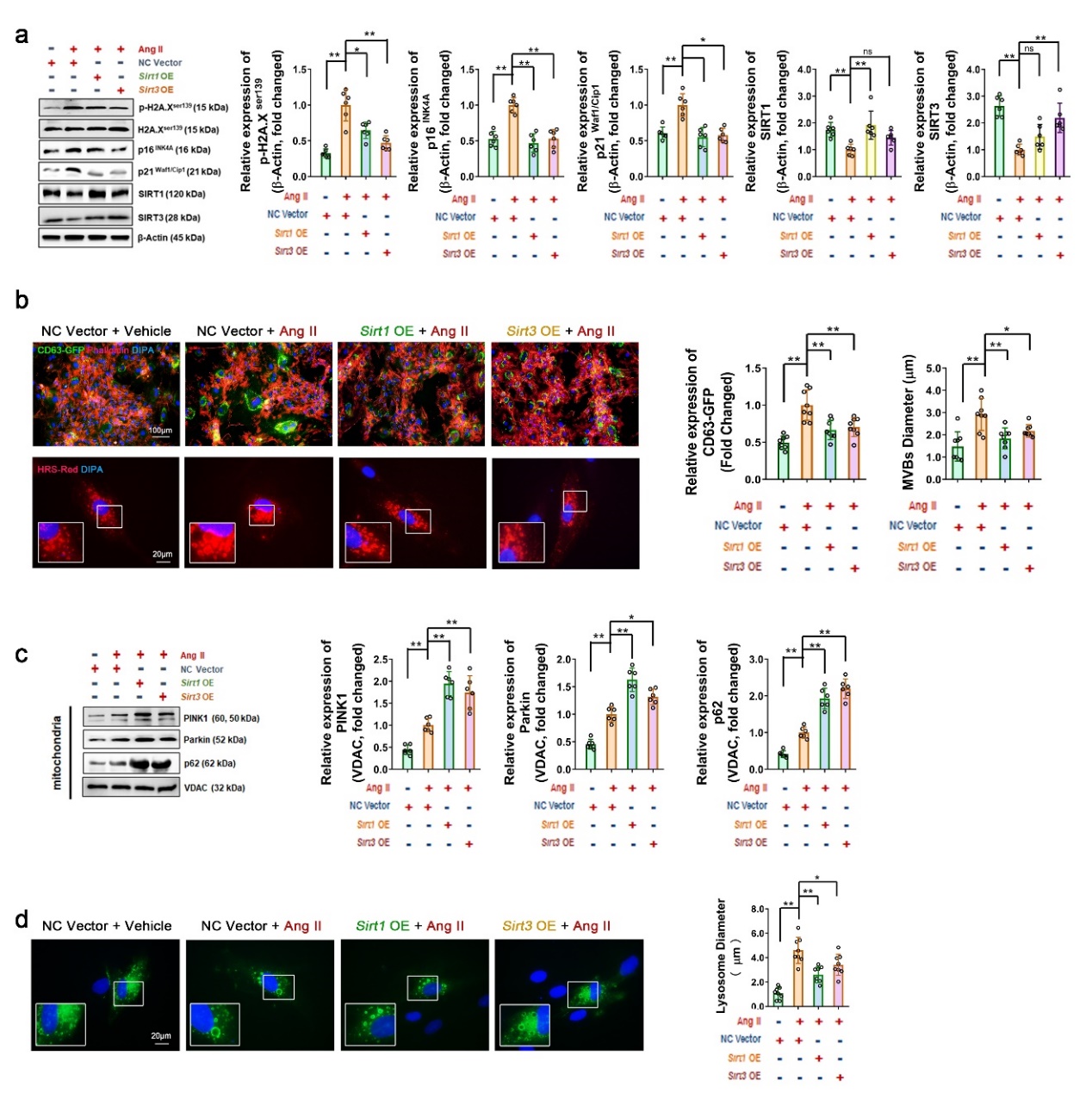
**

**Figure S8. SIRT1 and SIRT3 restored Ang II-induced VSMC senescence via inhibiting sEV biogenesis and promoting mitophagy. (a)** *In vitro* VSMC senescence analysis. The VSMCs transfected with NC vector or *Sirt1*/*Sirt3* plasmid and then treated with Ang II (100 nM). The senescence-associated proteins were detected by western blot analysis (n=6, One-Way ANOVA, **p*<0.05, ***p*<0.01). **(b)** *In vitro* sEV biogenesis analysis. The CD63-GFP-positive exosomes and HRS-RFP-positive MVBs were analyzed in stable WT CD63-GFP VSMCs. The VSMCs were transfected with NC vector or *Sirt1*/*Sirt3* plasmid plus pCS2 HRS-RFP plasmid and then treated with Ang II (100 nM) for 3 days. The exosomes and MVBs were tracked by green fluorescence and red fluorescence, respectively (n=8, One-Way ANOVA, **p*<0.05, ***p*<0.01). **(c)** *In vitro* mitophagy analysis in WT VSMCs overexpressing *Sirt1* and *Sirt3*. The VSMCs transfected with NC vector or *Sirt1*/*Sirt3* plasmid and then treated with Ang II (100 nM). The mitophagy-related proteins were detected by western blot analysis (n=6, One-Way ANOVA, **p*<0.05, ***p*<0.01). **(d)** *In vitro* lysosome function in VSMCs overexpressing *Sirt1* and *Sirt3*. The VSMCs transfected with NC vector or *Sirt1*/*Sirt3* plasmid plus LAMP1-mGFP plasmid and then treated with Ang II (100 nM) for 3 days. The green fluorescing structures were tracked to lysosomes (n=8, One-Way ANOVA, **p*<0.05, ***p*<0.01).
